# Phase 1 Study of Two Merozoite Surface Protein 1 (MSP1_42_) Vaccines for Plasmodium falciparum Malaria

**DOI:** 10.1371/journal.pctr.0020012

**Published:** 2007-04-06

**Authors:** Elissa Malkin, Carole A Long, Anthony W Stowers, Lanling Zou, Sanjay Singh, Nicholas J MacDonald, David L Narum, Aaron P Miles, Andrew C Orcutt, Olga Muratova, Samuel E Moretz, Hong Zhou, Ababacar Diouf, Michael Fay, Eveline Tierney, Philip Leese, Siddhartha Mahanty, Louis H Miller, Allan Saul, Laura B Martin

**Affiliations:** 1 Malaria Vaccine Development Branch, National Institute of Allergy and Infectious Diseases, National Institutes of Health, Rockville, Maryland, United States of America; 2 Biostatistics Research Branch, National Institute of Allergy and Infectious Diseases, National Institutes of Health, Bethesda, Maryland, United States of America; 3 PATH Malaria Vaccine Initiative, Bethesda, Maryland, United States of America; 4 Quintiles Phase 1 Services, Lenexa, Kansas, United States of America

## Abstract

**Objectives:**

To assess the safety and immunogenicity of two vaccines, MSP1_42_-FVO/Alhydrogel and MSP1_42_-3D7/Alhydrogel, targeting blood-stage Plasmodium falciparum parasites.

**Design:**

A Phase 1 open-label, dose-escalating study.

**Setting:**

Quintiles Phase 1 Services, Lenexa, Kansas between July 2004 and November 2005.

**Participants:**

Sixty healthy malaria-naïve volunteers 18–48 y of age.

**Interventions:**

The C-terminal 42-kDa region of merozoite surface protein 1 (MSP1_42_) corresponding to the two allelic forms present in FVO and 3D7 P. falciparum lines were expressed in *Escherichia coli,* refolded, purified, and formulated on Alhydrogel (aluminum hydroxide). For each vaccine, volunteers in each of three dose cohorts (5, 20, and 80 μg) were vaccinated at 0, 28, and 180 d. Volunteers were followed for 1 y.

**Outcome Measures:**

The safety of MSP1_42_-FVO/Alhydrogel and MSP1_42_-3D7/Alhydrogel was assessed. The antibody response to each vaccine was measured by reactivity to homologous and heterologous MSP1_42_, MSP1_19_, and MSP1_33_ recombinant proteins and recognition of FVO and 3D7 parasites.

**Results:**

Anti-MSP1_42_ antibodies were detected by ELISA in 20/27 (74%) and 22/27 (81%) volunteers receiving three vaccinations of MSP1_42_-FVO/Alhydrogel or MSP1_42_-3D7/Alhydrogel, respectively. Regardless of the vaccine, the antibodies were cross-reactive to both MSP1_42_-FVO and MSP1_42_-3D7 proteins. The majority of the antibody response targeted the C-terminal 19-kDa domain of MSP1_42_, although low-level antibodies to the N-terminal 33-kDa domain of MSP1_42_ were also detected. Immunofluorescence microscopy of sera from the volunteers demonstrated reactivity with both FVO and 3D7 P. falciparum schizonts and free merozoites. Minimal in vitro growth inhibition of FVO or 3D7 parasites by purified IgG from the sera of the vaccinees was observed.

**Conclusions:**

The MSP1_42_/Alhydrogel vaccines were safe and well tolerated but not sufficiently immunogenic to generate a biologic effect in vitro. Addition of immunostimulants to the Alhydrogel formulation to elicit higher vaccine-induced responses in humans may be required for an effective vaccine.

## Editorial Commentary

**Background:** Generally, adults living in parts of the world where malaria is common develop protective immunity against the parasite. This means they may get infected but not become ill as a result. However, there are individuals, such as pregnant women and children under the age of five, who are more likely to develop symptoms of malaria due to no (or reduced) natural immunity. A successful malaria vaccine would stimulate an individual's immune system to respond to the malaria parasite and prevent serious clinical disease. Many different groups are currently developing potential vaccines. Several candidates are based on a protein called MSP1 (merozoite surface protein 1) which is found on the surface of the blood-stage form of the malaria parasite. However, in nature parasites carry different versions of the MSP1 protein, and ideally a successful vaccine would bring about immune responses against these different versions. The researchers carrying out this trial wanted to compare the safety and immune responses against candidate vaccines representing two different MSP1 proteins, which covered many different parasite lines. As a phase 1 trial, the study was carried out in healthy adult volunteers. Sixty individuals were assigned to receive an injection of the vaccines, either containing a recombinant protein analogous to the FVO parasite line (termed MSP1_42_-FVO) or the 3D7 parasite line (termed MSP1_42_-3D7) at three different dose levels. The trial's primary objective was to assess safety, which was done by collecting data on any abnormal signs or symptoms up to 14 d after each of three vaccinations. These outcomes were graded and then defined as related to the vaccine or not. The researchers also looked at antibody levels in participants' blood against different variants of the MSP1 protein, as well as using in vitro tests to see whether antibodies from vaccinated individuals could prevent malaria parasites from growing in lab culture.

**What the trial shows:** The safety outcomes of the trial showed that the most common type of side effect experienced by the volunteers was pain at the injection site. The vast majority of such events were graded as mild, although there was one single case of a severe event (high levels of pain experienced by one volunteer at the injection site). There was no significant association between the chance of side effects and the vaccine dosage that an individual received. Following vaccination, antibody levels against the protein on which the vaccine was based were detected, although these levels dropped over time. The researchers did not see a strong association between the vaccine dosage that individuals received and the level of antibody response. However, the two vaccines when compared seemed to be equally good at raising an immune response and both caused antibodies to be raised corresponding to different variants of the MSP1 protein. However, the antibodies raised did not seem to be particularly effective at preventing malaria parasites from growing in lab culture.

**Strengths and limitations:** Strengths of this study include a comparison of three different dosage levels of the vaccines under study, as well as a comparison of two vaccines based on the same protein, representing different parasite lines. Limitations to the study include the small number of participants, which makes the trial underpowered to detect all but large differences in side effects between the groups being compared. A placebo arm was not included in the trial, so it is not possible to be sure that the numbers of side effects observed here can be attributed to the vaccines or not. Finally, the procedure for assigning individuals to the two different vaccines involved alternation, rather than true randomization, which could have minimized the risk of bias.

**Contribution to the evidence:** The trial reported here is an essential step in vaccine development. The results provide the first evidence relating to safety for these two vaccines, and do not raise any safety concerns at this stage. Although the vaccines raised an immune response, the antibodies raised did not seem to have much of an effect on malaria parasites in vitro. While these vaccines are safe, alternative MSP1 vaccine formulations anticipated to bring about a greater immune response will likely be studied before proceeding to field studies.

## INTRODUCTION

The Plasmodium falciparum parasite is responsible for at least 300 million cases of malaria each year [1], and more than one million of these cases result in death [2]. Approximately 90% of these deaths, the majority in children under 5 y of age, occur in Africa [3,4]. The clinical symptoms and pathology associated with P. falciparum infection are associated with the cyclical invasion of erythrocytes, intracellular parasite multiplication, and release of parasites by rupture of the infected cells. A vaccine that interrupted this cycle of infection could reduce both mortality and morbidity secondary to P. falciparum infection and would be a valuable resource in the fight against this disease.

Over time, people living in endemic areas develop immunity to severe disease due to P. falciparum as a result of repeated infection [5,6]. This acquired immunity is mediated, in part, by blood-stage parasite-specific antibodies [7–9]. Thus, parasite proteins expressed during the blood-stage have been proposed to be good candidates for inclusion in a vaccine. The aim of an asexual blood-stage vaccine is to elicit immune responses that slow or inhibit parasite multiplication to prevent morbidity, severe disease, and death in residents of malaria-endemic areas, primarily young children and infants.

*P. falciparum's* merozoite surface protein 1 (MSP1) is synthesized as a ∼200-kDa polypeptide. MSP1 is processed at, or just prior to, merozoite release from the erythrocyte into smaller fragments that form a noncovalently associated complex [10]. The C-terminal 42-kDa cleavage product of MSP1 (MSP1_42_), a major candidate for a blood-stage malaria vaccine, is composed of two regions: MSP1_33_ and MSP1_19_ [11]. The MSP1_33_ portion is dimorphic. Although the sequence similarity between the two forms of MSP1_33_ is surprisingly low (47% identity) within each dimorphic type, the sequence is highly conserved. The MSP1_19_ domain is largely conserved between parasite strains [12,13]. Four commonly observed amino acid substitutions have been identified in MSP1_19_ in laboratory lines with several additional polymorphisms in field isolates [13,14]. The MSP1_42_ proteins of the FVO and 3D7 P. falciparum parasite lines cover both dimorphisms in MSP1_33_ and the more common antigenic diversity in MSP1_19_. The inclusion of both MSP1_42_-FVO and MSP1_42_-3D7 proteins in a combination vaccine would, in large part, address the concerns of generating protective immune responses to a polymorphic parasite protein.

In studies of natural infection, the majority of the B cell epitopes have been localized to the highly conserved MSP1_19_ domain [15,16], and the epitopes that induce proliferation of T cell subsets specific for MSP1_42_ have been localized to the dimorphic region of the molecule MSP1_33_ [15,16]. A combination MSP1_42_-FVO and MSP1_42_-3D7 vaccine may be essential to prime for, or to boost immune responses, that would ultimately result in optimal immune responses in endemic areas.

Previous human trials have evaluated recombinant protein vaccines based on the C terminus of MSP1. The first trial evaluated fusion proteins of FVO and 3D7 forms of MSP1_19_ with the tetanus toxoid universal T cell epitopes P30P2 formulated on Alhydrogel in a United States population [17]. These vaccines were poorly immunogenic and their administration resulted in three immediate-type hypersensitivity reactions, which halted the trial. More recent trials assessed recombinant MSP1_42_-3D7 (FMP-1) formulated with GlaxoSmithKline Biologicals' proprietary adjuvant AS02A in the United States and Africa (Kenya and Mali) [18–21]. In all populations, FMP1/AS02A was safe and immunogenic. In the malaria-naïve population, biologically active antibodies and antigen reactive T cells were induced [19]. There have been no trials to date evaluating the immunogenicity of MSP1_42_-FVO or comparing the specificity of the human responses to the two dimorphic forms of MSP1_42_.

The Malaria Vaccine Development Branch (MVDB), National Institutes of Allergy and Infectious Diseases (NIAID), National Institutes of Health (NIH) manufactured two individual clinical grade recombinant MSP1_42_ proteins derived from the FVO and 3D7 parasite lines of P. falciparum with the ultimate aim of using them in a combination vaccine. Each MSP1_42_ protein was individually formulated on Alhydrogel (Brenntag Biosector, Denmark), an aluminum hydroxide gel, to produce the vaccines, MSP1_42_-FVO/Alhydrogel and MSP1_42_-3D7/Alhydrogel. This paper compares the safety and immunogenicity of these individual formulations when tested in a healthy United States adult population.

## METHODS

### Participants

Sixty healthy volunteers, 18–48 y of age, were recruited from the Lenexa, Kansas area. Written informed consent was obtained prior to enrollment. Volunteers were recruited and consented using a protocol and consent form approved by the Heartland Institutional Review Board (trial site IRB), the PATH Human Subjects Protection Committee, and the NIAID IRB. Volunteers were excluded if they had any of the following: evidence of clinically significant systemic disease; obesity (body mass index ≥35); pregnancy or breast-feeding; serological evidence of human immunodeficiency virus infection, chronic hepatitis B or hepatitis C infection; current medication with corticosteroids or immunosuppressive drugs; immunization with a live vaccine 4 wk prior to entry or a killed vaccine 2 wk prior to entry into the study; prior malaria infection; previous receipt of a malaria vaccine; travel to a malaria-endemic country 12 mo prior to study enrollment; or planned travel to a malaria-endemic country during the course of the study. All females had a urine ß human chorionic gonadotropin test at screening and immediately prior to each vaccination.

### Intervention: Purification and Characterization of Clinical Grade MSP1_42_ Antigens and Vaccine Preparation

The expression, refolding, and purification of *E. coli-*produced MSP1_42_-FVO (lot WRAIR0997) and MSP1_42_-3D7 (lot WRAIR0984) antigens was performed at the Walter Reed Army Institute of Research, Pilot Bioproduction Facility, Silver Spring, Maryland in accordance with current Good Manufacturing Practices (cGMP). Each protein underwent comprehensive quality control analysis to ensure purity, identity, and integrity. MSP1_42_-FVO and MSP1_42_-3D7 recombinant proteins are highly purified 42,173-Da and 44,236-Da polypeptides, respectively, which correspond to the external domain of MSP1_42_ from the P. falciparum FVO and 3D7 lines.

EcMSP1_42_-FVO and EcMSP1_42_-3D7 were each expressed as histidine-tagged fusion proteins from synthetic genes, cloned into pET-24d or pET-24a (Novagen, http://www.novagen.com), respectively, and transformed into BL21 (DE3) E. coli (Invitrogen, http://www.invitrogen.com). Following E. coli fermentation in defined medium and isopropyl-β-D-thiogalactopyranoside induction, each protein was extracted from solublized inclusion bodies, and EcMSP1_42_-FVO underwent an initial purification by reverse-phase chromatography. This product or the extracted EcMSP1_42_-3D7 was subjected to nickel-affinity chromatography, refolding by rapid dilution, and further purified by anion-exchange chromatography and size-exclusion chromatography (L. B. Martin, et al., unpublished data). Both antigens were supplied in sterile phosphate buffered saline (PBS [pH 7.4]) containing 0.2% polysorbate 80. For EcMSP1_42_-FVO, the final purity (percentage in a single band by SDS-PAGE and densitometry) was ∼95.0%, with a host cell protein impurity level of 0.036% and endotoxin level (as measured by the Limulus amoebocyte lysate gel clot assay) of 0.12 endotoxin units (EU)/mg. The results for EcMSP1_42_-3D7 were final purity ∼97.4%, host cell protein impurity level of 0.096%, and endotoxin level of 6.7 EU/mg.

Clinical grade MSP1_42_-FVO or MSP1_42_-3D7 recombinant protein was adsorbed to Alhydrogel and vialed by the Pharmaceutical Development Section, NIH. Three lots were prepared, containing 5, 20, or 80 μg of MSP1_42_-FVO or MSP1_42_-3D7, and 800 μg of Alhydrogel per 0.5 mL dose. The formulations were supplied as single-dose vials of a cloudy suspension, without additional stabilizers or preservatives, in a sterile saline solution. Each vaccine lot underwent comprehensive quality control analysis to ensure purity, identity, and integrity. The continued potency and stability of the six lots of vaccine stored at 2–8 °C were confirmed by evaluation of their immunogenicity in mice conducted every 6 mo throughout the course of the trial. Biochemical stability was evaluated annually.

### Intervention: Phase 1 Study Design

**Figure 1:**
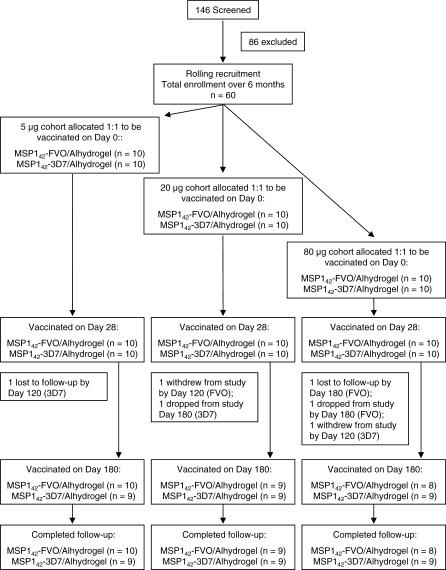
CONSORT Flow Chart Volunteers were enrolled following rolling-recruitment and assigned to one of six groups. Volunteers were alternately placed to receive either the MSP1_42_-FVO/Alhydrogel or MSP1_42_-3D7/Alhydrogel vaccine after assignment into a dose cohort. Volunteers were seen 1, 3, 7, and 14 d after each vaccination and on days 270 and 364 of the study. Immunologic assessment was carried out on samples obtained on study days 0, 14, 28, 42, 120, 180, 194, 270, and 364. 54 of 60 volunteers completed the entire study; three were lost-to-follow-up, one withdrew consent, and two did not receive the third vaccination and were followed for safety only.

An open-label, dose-escalating Phase 1 clinical trial was designed to evaluate the safety, reactogenicity, and immunogenicity of the MSP1_42_-FVO and MSP1_42_-3D7 recombinant proteins formulated on Alhydrogel in healthy adult volunteers. Thirty volunteers received the MSP1_42_-FVO/Alhydrogel vaccine and 30 volunteers received the MSP1_42_-3D7/Alhydrogel vaccine (Figure 1). Rolling recruitment and enrollment took place to fill the low-dose cohorts prior to the medium-dose cohort followed by the high-dose cohort. After enrollment to a dose cohort, volunteers were alternatively assigned to either the MSP1_42_-FVO/Alhydrogel or MSP1_42_-3D7/Alhydrogel vaccine group. Ten volunteers were assigned to each of three dose cohorts (5, 20, and 80 μg) for each vaccine for a total of six groups (three groups for MSP1_42_-FVO/Alhydrogel and three groups for MSP1_42_-3D7/Alhydrogel). Volunteers were vaccinated with a 0.5-mL intramuscular injection in alternate arms on study days 0, 28, and 180. Escalation to the next higher dose required approval by an independent safety monitoring committee.

This study was performed under an investigational new drug application (BB-IND Number 11635) approved by the United States Food and Drug Administration. MSP1_42_-FVO/Alhydrogel and MSP1_42_-3D7/Alhydrogel were considered separate candidate vaccines contained within one protocol. The protocol, amendments to the protocol, informed consent form, advertisements, and other study-related documents were approved by the Heartland Institutional Review Board (trial site IRB), the PATH Human Subjects Protection Committee, and the NIAID IRB.

### Objectives

The goal of this Phase 1 vaccine trial was to demonstrate safety and immunogenicity of MSP1_42_/Alhydrogel malaria vaccines in human volunteers. The primary objective was to determine the frequency and severity of vaccine-related adverse events for each dose of vaccine. Secondary objectives assessed and compared the specific antibody response to homologous and heterologous MSP1_42_ protein with time.

### Outcomes: Assessment of Safety and Tolerability

The primary outcome was to assess the safety and tolerability of the vaccines. Following each vaccination, the volunteers were observed for 60 min and then evaluated for evidence of local and systemic reactogenicity on days 1, 3, 7, and 14 after each vaccination. Solicited systemic adverse events included fever (>37.5 °C), headache, nausea, malaise, myalgia, arthralgia, and urticaria. Volunteers were asked to keep a diary card to record oral temperature and any local or systemic adverse events daily for 6 d following each vaccination. Diary cards were collected 7 d after each vaccination. An abbreviated history and physical examination was performed at each follow-up visit. All abnormal signs and symptoms were considered adverse events. Each adverse event was graded for severity and assigned causality relative to the study vaccine using the following terms: definite, probable, possible, remote, or unrelated. A complete blood count and white blood cell differential, as well as serum creatinine, aspartate aminotransferase, and alanine aminotransferase were performed immediately prior to each vaccination, as well as 3 and 14 d after each vaccination.

### Outcomes: Antibody Measurement by Enzyme-Linked Immunosorbent Assay

Secondary outcomes were enzyme-linked immunosorbent assay (ELISA) measurements of specific serum antibodies to the FVO and 3D7 forms of MSP1_42_, MSP1_19_, and MSP1_33_. A standardized ELISA protocol was employed for the measurement of anti-MSP1_42_, anti-MSP1_19_, and anti-MSP1_33_ antibodies, representing both the FVO and 3D7 forms of the proteins, as previously described [22]. A human anti-MSP1 standard was prepared by pooling sera from ten individuals residing in Mali, a country endemic for P. falciparum malaria. The standard serum pool was assigned 10,000 ELISA units on MSP1_42_-FVO, 5,882 ELISA units on MSP1_42_-3D7, 2,800 ELISA units on MSP1_19_-FVO, and 1,500 ELISA units on MSP1_19_-3D7. An ELISA unit was approximately equivalent to the reciprocal of the dilution giving an optical density (OD) of 1.0. A volunteer was considered to be a non-responder if a specific antibody level was less than 50 ELISA units. Due to the low titer in the standard serum pool, ELISA unit values for MSP1_33_ were not assigned.

The expression and purification of Saccharomyces cerevisiae-produced MSP1_19_ and E. coli-produced MSP1_33_ were carried out by MVDB. ScMSP1_19_–FVO and ScMSP1_19_-3D7 clones were fermented [23] and purified essentially as reported previously [24], except that the anion exchange step was excluded due to the low endotoxin levels of the yeast products. ScMSP1_19_-FVO and ScMSP1_19_-3D7 proteins were supplied in PBS.

E. coli BL21 (DE3) cells transformed with EcMSP1_33_-FVO or EcMSP1_33_-3D7 plasmids were fermented, inclusion bodies isolated, and the solubilized inclusion bodies were purified by nickel-affinity chromatography [24]. Due to the low solubility of EcMSP1_33_-FVO, it could not be effectively purified further and was supplied in elution buffer containing 4 M guanidine. EcMSP1_33_-3D7 was further purified by anion exchange chromatography and size-exclusion chromatography, as described for ScMSP1_19_. EcMSP1_33_-3D7 was supplied in PBS plus 0.2% polysorbate 80.

The purified ScMSP1_19_ and EcMSP1_33_ recombinant proteins were characterized by reverse-phase HPLC [25], N-terminal sequencing, electron-spray ionization mass spectrometry, SDS-PAGE (reduced and nonreduced), and immunoblot essentially as described [24]. The observed results were similar to those expected for each recombinant protein.

### Outcomes: Isotyping of MSP1_42_ Antibodies Using Suspension Array Technology

Serum samples from volunteers vaccinated with MSP1_42_-FVO/Alhydrogel or MSP1_42_-3D7/Alhydrogel were assayed for IgG subclasses against the immunizing antigen using a flow cytometric suspension array assay. Serum samples were diluted 1:100 and mixed with microspheres coupled with the homologous MSP1_42_ antigen (Luminex Corporation, http://www.luminexcorp.com) in MultiScreen plates (Millipore, http://www.millipore.com). Mouse anti-human IgG isotype antibody and a second donkey anti-mouse-IgG phycoerythrin (PE) labeled antibody (Jackson ImmunoResearch, http://www.jacksonimmuno.com) were added to develop the reaction. The mean fluorescence intensity (MFI), corresponding to the presence of specific IgG subclasses to MSP1_42_, was detected by Luminex X-MAP with the software Bioplex (Bio-Rad, http://www.bio-rad.com).

### Outcomes: In Vitro Parasite Growth Inhibition by Immune IgG and Immunofluorescence with Sera on Malaria Parasites

The ability of antibodies from vaccinated individuals to inhibit growth of P. falciparum FVO and 3D7 parasites in vitro was assessed using a standardized growth inhibition assay (GIA) as previously described [22]. To ensure that the inhibitory activity measured was specific to antibody and not other serum components, total IgG was purified from individual sera obtained on days 0, 42, and 194 using Protein G columns (Pierce, http://www.pierce.com). Anti-MSP1_42_-FVO and anti-MSP1_42_-3D7 ELISA units were determined for each purified IgG sample (10 mg/mL), and all samples were stored in small aliquots at 2–8 °C until tested.

Indirect immunofluorescence assay (IFA) of P. falciparum FVO or 3D7 parasitized red blood cells (RBC, 3%–7% parasitemia) stained using volunteer sera obtained on day 0 and day 194. Thin smears from bulk parasite cultures were prepared and stored at −80 °C. The FVO and 3D7 parasite smears were validated using mouse monoclonal antibodies 1G3 and 4H9/19 specific for MSP1_42_-FVO and MSP1_42_-3D7 proteins, respectively, and anti-mouse IgG FITC (Zymed, http://www.zymed.com). After fixation with methanol and blocking with 10% bovine serum albumin (BSA Fraction V; Sigma, http://www.sigmaaldrich.com), preimmune (day 0) and day 194 sera (diluted 1:100) were allowed to react with the parasite-infected RBC. A positive serum (diluted 1:400) and negative control (no sera) were included on each slide. Antibody bound to the parasite was detected with secondary antibody, anti-human IgG FITC (ICN/CAPPEL, Aurora, Ohio, United States). The slides were examined with an immunofluorescent microscope (Olympus, http://www.olympus.com) and photographed using a digital camera (Olympus), and the smears scored on staining pattern and fluorescence intensity: 0 = negative or diffuse staining; 1 = weak staining with characteristics of trophozoites, schizonts, or free merozoites; and 2 = bright staining with characteristics of trophozoites, schizonts, or free merozoites. The day 194 sera with positive immunofluorescence were compared with corresponding day 0 sera for specificity of reactions to the parasitized RBC.

### Sample Size

The sample size required in each treatment group was based on analysis of the human antibody responses to a number of malaria antigens that have been tested in clinical trials [26,27]. Based on the distribution of antibody responses for each of the antigens, a sample size of ten volunteers per dose cohort would permit detection of at least a 5-fold difference in antibody concentration between groups using a Mann-Whitney test, assuming a level of significance of 0.05 and a power of 0.80. Additionally, a group size of ten volunteers per dose gives 0.80 probability for detecting one or more serious or severe adverse events that occurred with a probability of 0.15 per volunteer.

### Randomization

This was an open-label, dose-escalating clinical trial and no randomization procedure was utilized to assign volunteers to dose cohort. Temporal staggering was used to assign volunteers to dose cohorts. Volunteers were alternately assigned to receive either the MSP1_42_-FVO/Alhydrogel or MSP1_42_-3D7/Alhydrogel vaccine after enrollment in a dose cohort. Dose-escalation occurred only after safety data up to and including day 35 post-first vaccination; 7 d post-second vaccination was reviewed by the safety monitoring committee.

### Statistical Methods

The frequency of adverse events was summarized and stratified by dose cohort. Dose effects (on adverse events and on antibody response) were tested for using exact two-sided Jonckheere-Terpstra tests, and comparisons across the vaccinations within each dose cohort were compared using McNemar's test (SAS version 9.1, SAS Institute, http://www.sas.com). Tests of paired ELISA results (e.g., comparing day 0 to day 42 responses or comparing homologous to heterologous responses) were done by the Wilcoxon signed rank test using all available pairs. Confidence intervals for paired ELISA results were done by paired *t*-test. For the GIA, confidence intervals were done using *t*-distributions. Either the UNISTAT statistical package (version 5.5) or R (version 2.4.0 of R, using the exactRankTests package, version 0.8–15) were used for these analyses, and *p-*values of less than or equal to 0.05 were considered significant.

## RESULTS

### Participant Flow

This study was conducted from June 2004 to November 2005. Sixty volunteers (26 male and 34 female) were enrolled. The age range was 18–48 y (median, 27). Nine volunteers (15%) identified themselves as African American, three (5%) as Hispanic, and 48 (80%) as Caucasian. Fifty-four volunteers received all three vaccinations as scheduled (Figure 1). No volunteer was withdrawn due to a vaccine-related adverse event.

### Outcomes and Estimations

#### Safety and tolerability data.

Both the MSP1_42_-FVO/Alhydrogel and the MSP1_42_-3D7/Alhydrogel vaccines were safe and well tolerated (Tables 1 and 2). The most common reported adverse event was pain at the injection site. The majority of injection site reactions were graded as mild (98% for the MSP1_42_-FVO vaccine and 94% for the MSP1_42_-3D7 vaccine), and none were graded as severe. Erythema, induration, and pruritus also occurred at low frequencies (10%–25%). The most common solicited systemic adverse events were headache and fatigue. No immediate hypersensitivity reactions were observed and no serious adverse events occurred that were attributed to the vaccine.

**Table 1:**
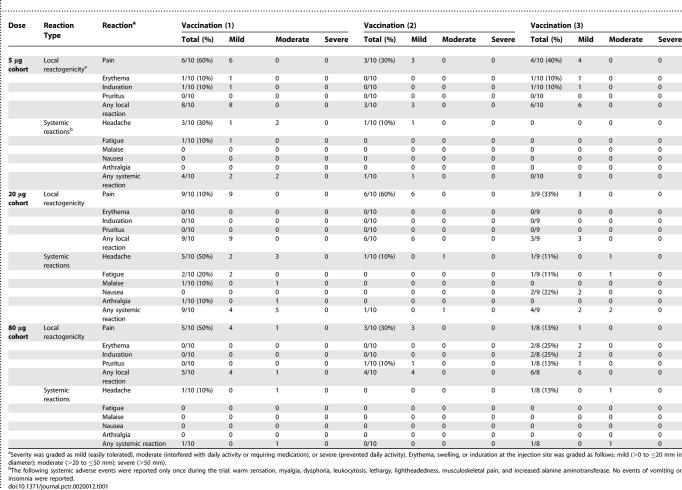
MSP1_42_-FVO/Alhydrogel Vaccine Adverse Events

**Table 2:**
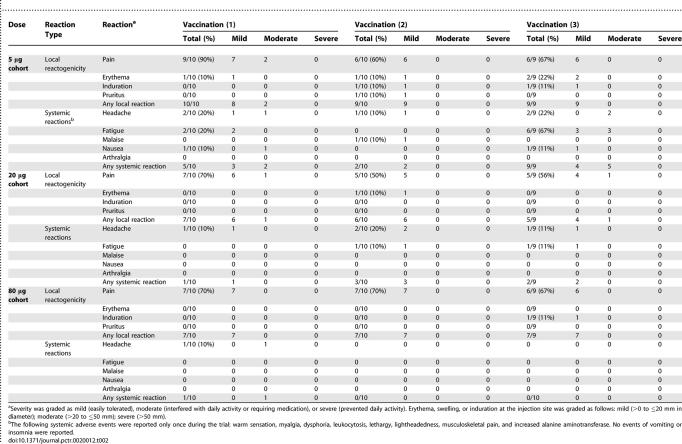
MSP1_42_-3D7/Alhydrogel Vaccine Adverse Events

No effect was found between dose and severity of the highest-grade adverse event observed for each volunteer (both vaccines: *p* = 0.762; MSP1_42_-FVO/Alhydrogel vaccine only: *p* = 0.764; or MSP1_42_-3D7/Alhydrogel vaccine only: *p* = 0.475). Additionally, no significant dose effect was found when looking at the number of local adverse events per volunteer relative to the dose (both vaccines: *p* = 0.601, MSP1_42_-FVO/Alhydrogel vaccine only: *p* = 0.594; or MSP1_42_-3D7/Alhydrogel vaccine only: *p* = 0.808). Local reactions were more frequent following the first vaccination compared to either the second (odds = 20:5, *p* = 0.004) or third vaccinations (odds = 21:5, *p* = 0.0025).

One volunteer in the 80-μg MSP1_42_-3D7/Alhydrogel vaccine group developed pain at the injection site following the third vaccination which lasted for 4 d. After the third vaccination, 8 d later, the volunteer complained of left upper extremity/shoulder pain which was rated as severe. The volunteer was evaluated in a local emergency room with a left upper extremity venous Doppler, plain film x-ray, and laboratory testing. The physical examination and test results were negative. The final diagnosis was an atypical injection site reaction/musculoskeletal pain based on the temporal association with vaccination and exclusion of other etiologies.

#### Antibody assessment by ELISA.

Antibody levels to both the MSP1_42_-FVO and MSP1_42_-3D7 antigens were measured by ELISA on sera obtained on days 0, 14, 28, 42, 120, 180, 194, 270, and 364. A subset of the sera samples were assessed for antibodies recognizing regions of the MSP1_42_ protein: anti-MSP1_19_-FVO and anti-MSP1_19_-3D7 antibodies (days 0, 42, 194, and 270) and anti-MSP1_33_-FVO and anti-MSP1_33_-3D7 antibodies (day 0 and 194).

**Figure 2:**
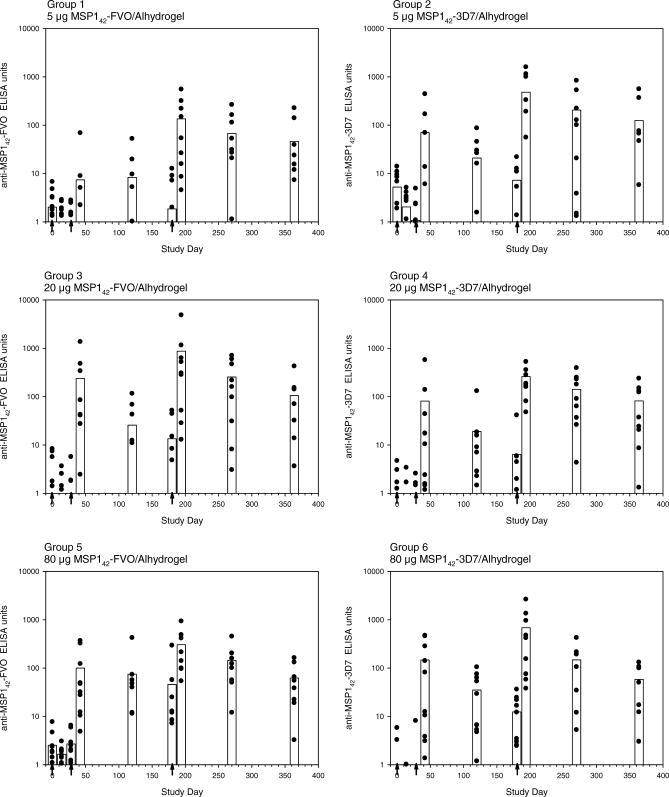
Kinetics of Homologous Anti-MSP1_42_ Antibody Responses Following MSP1_42_-FVO/Alhydrogel (Left) and MSP1_42_-3D7/Alhydrogel (Right) Vaccination Antibody ELISA units to the immunizing antigen for each individual are shown by the scatter plots. The bars represent the group arithmetic means. Volunteers were vaccinated intramuscularly on days 0, 28, and 180 (indicated by arrows) with 5 μg (top), 20 μg (middle), or 80 μg (bottom) dose of vaccine. ELISA units are expressed relative to a MSP1 standard serum pool. A response was defined as >50 ELISA units.

Figure 2 shows the anti-MSP1_42_ antibodies to the homologous protein in each dose of the MSP1_42_-FVO/Alhydrogel and MSP1_42_-3D7/Alhydrogel vaccinated groups. Elevated MSP1_42_-specific antibody levels were observed 2 wk following the second vaccination (day 42), when compared to prevaccination levels (*p* < 0.001 for both vaccines tested on either homologous or heterologous antigen). These antibodies diminished over time and in many volunteers had returned to near background levels by the time of the third vaccination. After the third vaccination (day 194), 2 wk later, a recall response was observed with an increase in specific anti-MSP1_42_ antibodies to levels greater than or equal to those observed after two vaccinations (*p* < 0.001 for both vaccines tested on either homologous or heterologous antigen). The elevated MSP1_42_-specific antibody levels observed on day 194 diminished with time, but 6 mo after the third vaccination, low-level specific antibody was still detectable.

Specific antibody levels following the third vaccination were greater than those observed after the second vaccination for all dose cohorts. The number of responders in all groups also increased with eight of 30 and ten of 30 responders after two vaccinations and 20 of 27 and 22 of 27 responders after three vaccinations with MSP1_42_-FVO/Alhydrogel and MSP1_42_-3D7/Alhydrogel, respectively. The effect of vaccine dose on specific antibody responses to either MSP1_42_-FVO or the MSP1_42_-3D7 protein on day 42 and 194 was evaluated for both of the MSP1_42_ vaccines. The day 42 ELISA of MSP1_42_-FVO/Alhydrogel on the MSP1_42_-FVO antigen was the only significant dose effect (*p* = 0.044), and this effect was only just significant. For the remainder of the comparisons, no significant correlation between vaccine dose and antibody response was detected.

**Figure 3:**
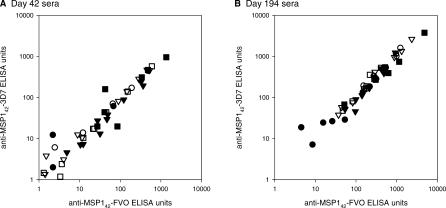
Comparison of MSP1_42_-FVO and MSP1_42_-3D7 Antibody Levels on (A) Day 42 and (B) Day 194 Sera from MSP1_42_-FVO/Alhydrogel and MSP1_42_-3D7/Alhydrogel Vaccinated Individuals Data for individual volunteers are shown by the scatter plots: 5-μg dose (circles), 20-μg dose (squares), and 80-μg dose (diamonds); closed symbols are the MSP1_42_-FVO/Alhydrogel vaccine and open symbols are the MSP1_42_-3D7/Alhdyrogel vaccine. Volunteers were vaccinated IM on days 0, 28, and 180 with the indicated dose of vaccine. ELISA units are expressed relative to a MSP1 standard serum pool.

The MSP1_42_-FVO/Alhydrogel and MSP1_42_-3D7/Alhydrogel vaccines were equally immunogenic. Among recipients of either vaccine, there was some evidence of MSP1_42_ strain specificity in the antibody response of sera from days 42 or 194 (Figure 3); sera recognized the homologous (i.e., immunizing) antigen better than heterologous antigen at day 194 (*p* < 0.001) but not at day 42 (*p* = 0.642). However, in both cases the mean absolute difference in ELISA units is small (day 42: −3 units, 95% confidence interval: −19 to 13; day 194: 58 units, 95% confidence interval: 10 to 106).

**Figure 4:**
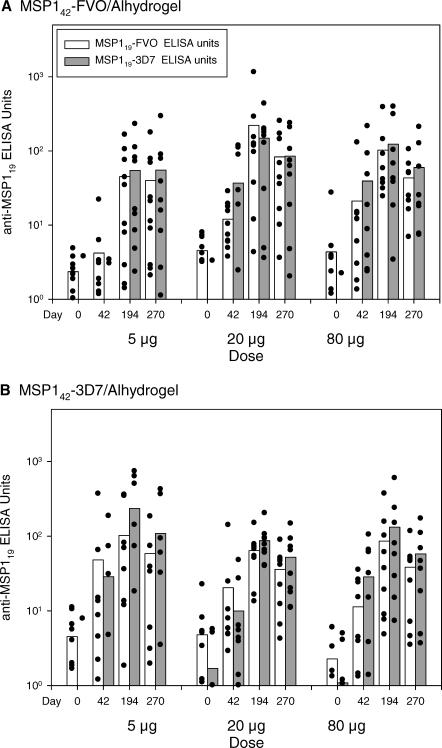
Homologous and Heterologous Anti-MSP1_19_ Antibody Levels Following MSP1_42_-FVO/Alhydrogel (A) and MSP1_42_-3D7/Alhydrogel (B) Vaccination Antibody ELISA units to the immunizing and heterologous forms of MSP1_19_ for each volunteer are shown by the scatter plot. The bars represent the group arithmetic means. Volunteers were vaccinated IM on days 0, 28, and 180 with 5-, 20-, or 80-μg dose of the indicated vaccine. ELISA units are expressed relative to a MSP1 standard serum pool.

Vaccination with MSP1_42_ generated antibodies that recognized the C-terminal domain of the MSP1_19_ protein. The antibodies specific to MSP1_19_ were observed following the second vaccination (day 42), increased following the third vaccination, and remained detectable 90 d after the third vaccination (day 270) (Figure 4). The only significant relationship between dose and anti-MSP1_19_ antibody levels was for the MSP1_42_-FVO/Alhydrogel vaccine at day 42 (*p* = 0.0238 when tested on MSP1_19_-FVO protein and *p* = 0.0378 on MSP1_19_-3D7 protein).

By day 42, individuals vaccinated with MSP1_42_-FVO/Alhydrogel or MSP1_42_-3D7/Alhydrogel generated similar levels of antibodies that recognized the homologous and heterologous MSP1_19_ proteins (Wilcoxon signed rank test, *p* > 0.05). Comparison of the MSP1_19_ antibody reactivity in sera from days 194 and 270 of the MSP1_42_-FVO vaccine showed no difference between homologous and heterologous MSP1_19_ proteins. But within the MSP1_42_-3D7 vaccine, individual sera collected on days 194 and 270 recognized MSP1_19_-3D7 (homologous) protein better than MSP1_19_-FVO (heterologous) protein (Wilcoxon signed rank test, *p* < 0.004 for each day); the absolute difference in ELISA units was very small. For both vaccines, a strong association between MSP1_42_ antibodies and MSP1_19_ antibodies was observed after day 194 (Spearman rank correlation: *r* > 0.94 and *p* < 0.0001 for all homologous comparisons on days 194 and 270).

**Figure 5:**
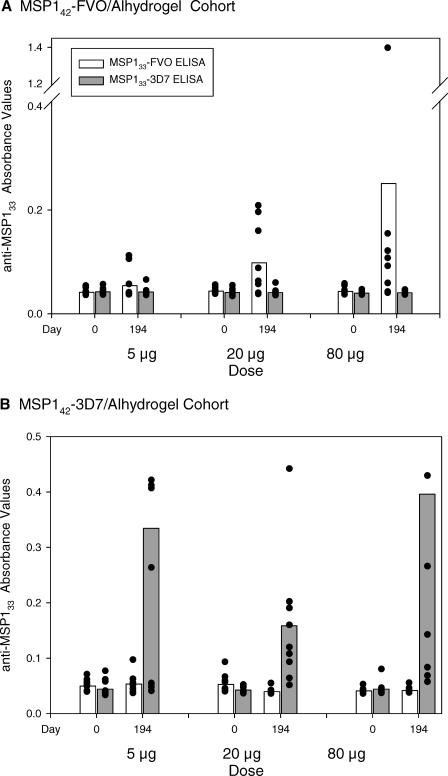
Homologous and Heterologous Anti-MSP1_33_ Antibody Levels Following Three Vaccinations with MSP1_42_-FVO/Alhydrogel (A) and MSP1_42_-3D7/Alhydrogel (B) Optical density in the standard ELISA assay to the immunizing and heterologous form of MSP1_33_ for each volunteer serum diluted 1:500 is shown by the scatter plots. The bars represent the group arithmetic means. Volunteers were vaccinated IM on days 0, 28, and 180 with 5-, 20-, or 80-μg dose of the indicated vaccine.

Following three vaccinations (day 194), a small increase in antibodies to the homologous MSP1_33_ protein was observed in both the MSP1_42_-FVO/Alhydrogel and MSP1_42_-3D7/Alhydrogel vaccinated groups (Figure 5). Increased antibody response to the heterologous MSP1_33_ protein was not observed. There was no correlation between vaccine dose and homologous anti-MSP1_33_ antibody levels. For both vaccines, a significant association between homologous MSP1_42_ and MSP1_33_ antibodies was observed (MSP1_42_-3D7/Alhydrogel Spearman rank correlation, *r* = 0.89, *p* < 0.0001 and MSP1_42_-FVO/Alhydrogel Spearman rank correlation, *r* = 0.78, *p* < 0.0001). A correlation was also observed between homologous anti-MSP1_33_ and anti-MSP1_19_ antibodies (Spearman rank correlation: FVO proteins, *r* = 0.73, *p* < 0.0001 and 3D7 proteins, *r* = 0.84, *p* < 0.0001).

#### IgG subclass analysis of day 194 sera.

**Figure 6:**
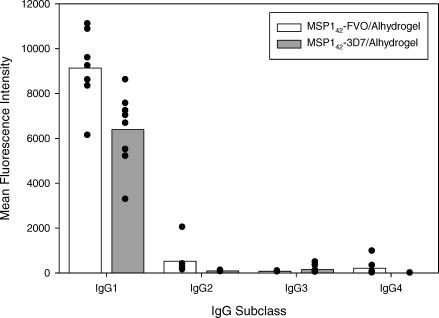
IgG Subclass Analysis of Antibodies Generated by Vaccination with MSP1_42_-FVO/Alhydrogel and MSP1_42_-3D7/Alhydrogel Day 194 serum samples from seven volunteers vaccinated with MSP1_42_-FVO/Alhydrogel and eight with MSP1_42_-3D7/Alhydrogel were assayed for IgG subclasses against the immunizing antigen. Serum samples were diluted 1:100 and mixed with beads coupled with homologous MSP1_42_ antigen. Mouse anti-human IgG isotypes and donkey anti-mouse-IgG PE labeled was added to develop the reaction. The mean fluorescence intensity of specific IgG subclasses to MSP1_42_ is presented.

Sera obtained at day 194 with MSP1_42_-specific antibody units greater than 500 were analyzed by a multiplex Luminex system for the proportion of MSP1_42_-specific IgG1, IgG2, IgG3, and IgG4 antibodies. For volunteers vaccinated with either MSP1_42_-FVO/Alhydrogel (*n* = 7) or MSP1_42_-3D7/Alhydrogel (*n* = 8), the majority of the MSP1_42_ antibodies were IgG1 (Figure 6), consistent with the IgG subclass profile of an Alhydrogel-based vaccine.

#### Parasite GIA.

The GIA is designed to measure the biologic function of the antibodies by determining whether anti-MSP1_42_ antibodies can inhibit merozoite invasion into erythrocytes. Analysis of preclinical immunogenicity studies in a variety of animals has shown a positive correlation between anti-MSP1_42_ antibody levels and inhibition of parasite growth in vitro [28] (L. B. Martin, et al., unpublished data). Immunoglobulin G (IgG) was purified from day 0 and 42 sera of the 80-μg dose cohorts. Subsequently, IgG was also purified from days 180 and 194 sera of all volunteers with an anti-MSP1_42_ ELISA level on day 194 greater than 500 antibody units (*n* = 7 from the MSP1_42_-FVO/Alhydrogel and *n* = 8 from the MSP1_42_-3D7/Alhydrogel). The IgG was tested for activity in the GIA using both the FVO and 3D7 P. falciparum lines.

None of the IgG samples purified from volunteer sera either on day 42 or 194 showed growth inhibitory activity higher than 20% (unpublished data) when tested at an IgG concentration in the assay of 2.5 mg/mL (approximately 25% of in vivo levels). Purified IgG from day 194 samples with an ELISA concentration of >500 units were also tested at a concentration of 5 mg/mL (ELISA units in the assay ranged from 200–1,400 units) and there was small but significant activity (GIA on FVO parasites: mean 11.9, 95% confidence interval: 9.4 to 14.4; GIA on 3D7 parasites mean: 3.6, 95% confidence interval 2.1 to 5.1). Significant correlations were obtained between the level of anti-MSP1_42_ antibody on day 194 and percent growth inhibition of both the FVO and 3D7 parasite lines (Spearman rank correlation, *r* = 0.56, *p* = 0.0177 and *r* = 0.8394, *p* = 0.0001, respectively).

#### Parasite IFA.

The ability of 53 of 54 volunteer's sera obtained at day 0 and day 194 to recognize the native MSP1 protein on P. falciparum FVO and 3D7 parasites was evaluated. Sera from all individuals (diluted 1:100) were evaluated for intensity of staining and the staining pattern of parasitized RBC.

Of the samples tested, minimal diffuse fluorescence was detected in the day 0 serum samples. After three vaccinations with MSP1_42_-FVO/Alhydrogel or MSP1_42_-3D7/Alhydrogel, half of the day 194 serum samples (27 of 54 sera on FVO parasites and 24 of 54 on 3D7 parasites) exhibited positive fluorescence staining of trophozoites (characteristic surface pattern) and schizont (morphology of grape-like clusters) blood-stage parasites as well as surface pattern of free merozoites (unpublished data). Regardless of the vaccine, the immune sera recognized the homologous and the heterologous native parasite antigen similarly (Wilcoxon signed rank test, *p* = 0.6509). Intensity of staining in the IFA correlated with MSP1_42_ ELISA units at day 194 (Spearman rank correlation, *r* = 0.7628 with *p* < 0.0001 for FVO parasites and ELISA, and *r* = 0.7726 with *p* < 0.0001 ELISA for 3D7 parasites and ELISA).

## DISCUSSION

### Interpretation

This study compared the safety and immunogenicity of two dimorphic forms of the MSP1_42_ antigen in humans. The results of this trial demonstrate both MSP1_42_-FVO/Alhydrogel and MSP1_42_-3D7/Alhydrogel are safe when administered to healthy adult malaria-naïve volunteers. The safety profile of these blood-stage vaccines supports the further development of a vaccine containing both proteins, referred to as MSP1_42_-C1.

An important secondary objective was to compare the ability of the MSP1_42_-FVO and MSP1_42_-3D7 vaccines to elicit specific antibody responses in malaria-naïve individuals after primary immunization and revaccination. For both MSP1_42_-FVO/Alhydrogel and MSP1_42_-3D7/Alhydrogel groups, no antibody response to the homologous immunogen was detected 2 wk after the first vaccination, but an increase in specific antibody was observed in volunteers after the second and third immunizations of either vaccines. These observations suggest that the initial immunizations induced B cell memory and revaccination boosted memory B cell responses. The proportion of responders in all dose cohorts increased markedly after each vaccination.

### Generalizability

MSP1_42_-FVO and MSP1_42_-3D7 polypeptides provide multiple B cell and T cell epitopes [15,16], and their amino acid compositions differ by greater than 50%, predominantly in the N-terminal 33-kDa region. The highly conserved C-terminal 19-kDa domain of MSP1_42_ is preferentially recognized by antibodies. Antibodies to MSP1_19_ prevent invasion and growth of the parasites in erythrocytes [29–31], and are also associated with protection against severe malaria [32–37]. Several T cell epitopes have been mapped to the dimorphic N-terminal 33-kDa region [16].

Preclinical studies showed overall antibody responses to MSP1_42_-FVO were greater than MSP1_42_-3D7. Although antibodies generated in response to vaccination cross-reacted with both forms of the protein, they preferentially recognized the homologous protein by ELISA and the homologous parasite by GIA (L. B. Martin, et al., unpublished data). In contrast, data from this clinical trial showed MSP1_42_-FVO/Alhydrogel and MSP1_42_-3D7/Alhydrogel were equally immunogenic, and the antibodies elicited by each vaccine recognized the homologous and heterologous MSP1_42_ recombinant proteins and native parasite proteins qualitatively similarly.

Antibody levels to MSP1_42_ protein correlated with antibody levels to both the MSP1_19_ and to MSP1_33_ proteins. These data indicate that both the conserved 19-kDa domain and the dimorphic 33-kDa region of MSP1_42_ are recognized by B cells and stimulate antibody responses. However, the majority of the antibodies induced by vaccination were targeted to the conserved domain, MSP1_19_. The biological impact of the low levels of anti-MSP1_33_ antibodies to the homologous protein is unknown. These data suggest that there is little difference in the antibody response generated by the dimorphic forms of MSP1_42_. The data from this study are in contrast to that obtained following vaccination with FMP1/AS02A where the resulting antibodies preferentially recognized the homologous antigen in malaria-naïve [19] and malaria-experienced [20,38] adults.

The nature of the MSP1_42_-specific antibody response and T cell immune response generated in the volunteers of the current study were found to be qualitatively different. Antibodies generated following vaccination with either MSP1_42_-FVO/Alhydrogel or MSP1_42_-3D7/Alhydrogel were highly cross-reactive in their ability to recognize the FVO or 3D7 form of MSP1_42_ and MSP1_19_, whereas the low-level antibody to the dimorphic MSP1_33_ region as allele-specific. This suggests limited strain specificity in the majority of the antibody response. In contrast, cytokine ELISPOT analysis of MSP1_42_-specific T cell responses revealed a strong preference toward the immunizing antigen with limited activation by the alternate form of MSP1_42_ (C. Huaman, et al., unpublished data). The epitopes responsible for the cytokine production were localized to the N-terminal 33-kDa region of MSP1_42_. This suggests that the development of a memory response following MSP1_42_ vaccination may require the inclusion of the relevant T-helper epitopes from the dimorphic region.

While there is considerable experimental support for the choice of MSP1_42_ as a vaccine candidate, the selection of this protein is potentially complicated by the dimorphism observed in different field isolates [39]. The levels of cellular and humoral immune responses required to protect against severe malaria are unknown. Thus, the requirements for a blood-stage vaccine to sensitize and potentiate appropriate immune responses that would protect individuals, particularly children, against malaria in areas of varying endemicity are even more speculative. Dramatically different immune responses have been reported in adult volunteers vaccinated with FMP1/AS02A in Mali and Kenya, areas of Africa that vary in parasite transmission [21]. This suggests that in a malaria-endemic area where multiple allelic forms of the parasite are present, a combination MSP1_42_-FVO and MSP1_42_-3D7 vaccine (MSP1_42_-C1) to prime for or boost immune responses may be required.

### Overall Evidence

The anti-MSP1_42_ antibodies induced by either MSP1_42_ vaccine recognized the native parasite protein, as shown by immunofluorescence microscopy, but did not give high levels of activity in the in vitro parasite growth inhibition assay at the concentrations tested. Vaccine-induced MSP1_42_ antibodies from preclinical studies in animals have shown substantial GIA activity, but generation of this activity requires either multiple vaccinations or formulation with other adjuvants [28,40] (L. B. Martin, et al., unpublished data). Biochemical stability studies and in vivo potency studies conducted over the course of the clinical trial have confirmed that the antigens in each of the formulations retained conformation. Therefore, it was concluded that the two MSP1_42_/Alhydrogel formulations are not sufficiently immunogenic to generate an antibody response able to block parasite invasion of erythrocytes detectable by the in vitro growth inhibition assay. On the basis of the correlation observed, it is estimated that at least a 10-fold higher antibody response will be needed before substantial GIA activity will be observed. Thus, if in vitro parasite GIA is found to be a good predictor of vaccine-induced protection of infants and children from malaria, then enhancing the immunogenicity of Alhydrogel-formulated MSP1_42_ will be an important step toward developing a useful vaccine. MVDB is pursuing the addition of immunostimulants to the Alhydrogel formulation of MSP1_42_-C1, the combination of MSP1_42_-FVO and MSP1_42_-3D7, to elicit higher immune responses in humans.

## SUPPORTING INFORMATION

CONSORT Checklist Click here for additional data file. (50 KB DOC)

Trial Protocol Version 7.1 (601 KB DOC)

## Accession Numbers

EcMSP1_42_-FVO (AY343089) and EcMSP1_42_-3D7 (DQ414722) were expressed as histidine-tagged fusion proteins from synthetic genes from GenBank (http://www.ncbi.nlm.nih.gov/Genbank). Also included are plasmids EcMSP1_33_-FVO (DQ923123) and EcMSP1_33_-3D7 (DQ923124).

## Abbreviations

ELISA: enzyme linked immunosorbant assay
GIA: growth inhibition assay
IFA: indirect immunofluorescence assay
MSP1: merozoite surface protein 1
MSP1_19_: C-terminal domain of MSP1_42_
MSP1_33_: N-terminal region of MSP1_42_
MSP1_42_: 42 kDa C-terminal region of MSP1
