# Supplementary material for: Phase 1 Study of Two Merozoite Surface Protein 1 (MSP142) Vaccines for Plasmodium falciparum Malaria
Source: PLoS Clin Trials. 2007 Apr 6;2(4):e12. doi: 10.1371/journal.pctr.0020012 (PMC1847697; doi:10.1371/journal.pctr.0020012)
Supplement: Trial Protocol — (601 KB DOC) [file pctr.0020012.sd002.pdf]

Phase 1 Study of the Safety and Immunogenicity of $MSP1_{42}$-FVO/Alhydrogel® and $MSP1_{42}$-3D7/Alhydrogel®, Asexual Blood-Stage Vaccines for *Plasmodium falciparum* Malaria

Version:	Version 7.0 (June 8, 2005)
Quintiles Protocol Number:	3532
NIAID Protocol Number:	04-I-N216
HSPC Protocol Number:	HS 229
FDA IND Number	BB-IND #11635
Ionizing Radiation:	None
Multi-institution:	No
Project Assurance:	FWA 00003974
Proposed start date:	May 2004
Proposed end date:	September 2005

<u>Sponsored by:</u>
Regulatory Compliance and Human Subjects Protection Branch (RCHSPB)
Office of Clinical Research (OCR), Office of the Director (OD)
National Institute of Allergy and Infectious Diseases (NIAID)
National Institutes of Health (NIH)

The information contained herein is the property of the National Institute of Allergy and Infectious Diseases and may not be reproduced, published or disclosed to others without the written authorization of the National Institute of Allergy and Infectious Diseases.

Team Roster

**Principal Investigator:**	Philip Leese, MD<br>Quintiles Phase 1 Services<br>11250 Corporate Avenue<br>Lenexa, KS 66219<br>(913) 752-8660
**Co-Investigators:**	
Malaria Vaccine Development Branch (MVDB)	Siddhartha Mahanty, MD<br>David Diemert, MD<br>National Institute of Allergy and Infectious Diseases (NIAID), National Insitutes of Health (NIH)<br>5640 Fishers Lane, Twinbrook I<br>Rockville, MD 20852<br>Tel/Fax: (301) 435-4305; (301) 480-1962
**Scientific Investigators:**<br>MVDB	Laura Martin, PhD<br>Louis Miller, MD<br>Allan Saul, PhD<br>Carole Long, PhD
**Funding Sponsor:**<br>Malaria Vaccine Inititative (MVI) at the Program for Appropriate Technology in Health (PATH)	Eveline Tierney, BS<br>Filip Dubovsky, MD<br>Elissa Malkin, DO, MPH<br>7500 Old Georgetown Road, 12th floor<br>Bethesda, MD 20814<br>Tel/Fax: (240) 395-2700; (240) 395-2591
**IND Sponsor Representative:**	John Tierney, RN<br>RCHSPB, OCR, OD<br>NIAID, NIH<br>6700-B Rockledge Drive, MSC 7609<br>Bethesda, MD 20892-7609<br>(301) 451-5136
**Local Safety Monitor:**	Bruce Short, MD<br>10550 Quivira<br>Suite 510<br>Overland Park, KS 66215<br>Tel/Fax: (913) 541-3340; (913) 492-7857

Protocol Statisticians:

Michael Fay, PhD
Biostatistics Research Branch
NIAID/NIH
6700A Rockledge Drive, Room 5133
Bethesda, MD 20892
(301) 451-5124

Participating Sites

Clinical Trial Site:

Quintiles Phase 1 Services
11250 Corporate Avenue
Lenexa, KS 66219
(913) 752-8660

Participating Laboratories

Immunology Laboratory:

Malaria Vaccine Development Branch
NIAID, NIH
5460 Fishers Lane, Twinbrook I
Rockville, MD 20852
(301) 435-3405

Clinical Laboratory:

Physicians Reference Laboratory
7800 West 110th Street
Overland Park, KS 66210
(913) 338-4070

Table of Contents

Team Roster ... 2
Protocol Summary ... 7
1.0 Introduction ... 8
1.1 Background ... 8
1.1.1 $MSP1_{42}$ Vaccines ... 9
1.2 Vaccine Description ... 9
1.2.1 $MSP1_{42}$ Drug Substances ... 9
1.2.2 Alhydrogel® ... 9
1.2.3 Vaccine Formulation, Drug Products ... 10
1.3 Rationale ... 10
1.3.1 Preclinical Experience with $MSP1_{42}$-FVO and $MSP1_{42}$-3D7 ... 10
1.3.2 Clinical Experience with $MSP1_{42}$ Vaccines ... 10
1.3.3 Clinical Experience with Aluminum-Based Adjuvants ... 11
1.4 Clinical Development Plan ... 11
1.4.1 Participation of Children ... 12
2.0 Objectives ... 12
2.1 Primary Objective ... 12
2.2 Secondary Objectives ... 12
3.0 Study Design ... 12
3.1 Overall Design ... 12
3.2 Sample Size and Estimated Duration of Study ... 13
3.3 Group Allocation ... 13
4.0 Selection and Enrollment of Volunteers ... 14
4.1 Inclusion Criteria ... 14
4.2 Exclusion Criteria ... 14
4.3 Treatments That Could Potentially Interfere with Vaccine-Induced Immunity .. 15
4.4 Contraindications to Vaccination ... 15
4.5 Indications for Deferral of Vaccination ... 15
4.6 Subject Withdrawal Criteria ... 16
5.0 Vaccine Preparation ... 16
5.1 Supplies ... 16
5.2 Vaccine Storage ... 17
5.3 Vaccine Accountability ... 17
5.4 Disposition of Used/Unused Supplies ... 17
6.0 Study Procedures ... 17
6.1 Screening (Up to 60 Days Prior to Vaccination) ... 17
6.2 Enrollment ... 18
6.3 Immunization Procedure ... 18
6.4 Clinical Monitoring and Evaluation ... 18
6.5 Volunteer Symptom Diary ... 21
6.6 Laboratory Testing ... 22
6.7 Immunologic Testing ... 22
6.7.1 Antibody Assay (ELISA) ... 22
6.7.2 Growth Inhibition Assay (GIA) ... 23
7.0 Adverse Events Monitoring and Reporting ... 23
7.1 Definitions ... 23
7.1.1 Adverse Event (AE) ... 23
7.1.2 Serious Adverse Event (SAE) ... 23
7.2 Assessment of Adverse Events ... 24
7.2.1 Identification of AEs ... 24

7.2.2 Determination of Severity 24
7.2.3 Association with Receipt of the Study Vaccine 25
7.3 Adverse Event Reporting 26
7.4 Adverse Event Monitoring 26
7.4.1 Local Safety Monitor 26
7.4.2 Safety Monitoring Committee 26
7.5 Stopping Criteria 27
8.0 Data Collection and Monitoring 27
8.1 Source Documentation 27
8.2 Study Documentation 27
8.3 Retention of Records 28
8.4 Protocol Revisions 28
8.5 Clinical Investigator's Brochure 28
8.6 Study Monitoring 28
9.0 Statistical Considerations 29
9.1 General Design 29
9.1.1 Description of the Statistical Methods to Be Employed 29
9.1.2 Safety 31
9.1.3 Immunogenicity Analysis 31
9.2 Sample Size 31
10.0 Protection of Human Subjects 31
10.1 Institutional Review Board/Ethics Committee 31
10.2 Informed Consent 32
10.3 Risks 32
10.3.1 Venipuncture 32
10.3.2 Immunization 32
10.4 Benefits 33
10.5 Confidentiality 33
10.6 Compensation 33
11.0 References 31

Appendix A - Summary of Preclinical Studies for $MSP1_{42}$-FVO & $MSP1_{42}$-3D7 37
Appendix B - Malaria Vaccine Trial Print Advertisement 38
Appendix C - Malaria Vaccine Trial Radio Advertisement 39
Appendix D - Pre-enrollment Phone Screen 40
Appendix E - Schedule of Visits 41
Appendix F - Malaria Vaccine Side Effect Diary Card 42
Appendix G – Toxicity Table for Grading Laboratory Adverse Events 45
Appendix H – Common Toxicity Criteria for Grading Unexpected Adverse Events 47

PRINCIPAL INVESTIGATOR'S STATEMENT:

I, the undersigned, have reviewed this protocol, including appendices, and will conduct the clinical study as described and will adhere to the principles of the current Good Clinical Practices as well as the applicable U.S. FDA regulatory requirements. I have read and understood the contents of the Investigator's Brochure provided by the Malaria Vaccine Development Branch of the National Institutes of Allergy and Infectious Diseases at the National Institutes of Health.

Philip Leese, M.D.

____
Signature

____
Date

Protocol Summary

Protocol Title: Phase 1 Study of the Safety and Immunogenicity of $MSP1_{42}$-FVO/Alhydrogel® and $MSP1_{42}$-3D7/Alhydrogel®, Asexual Blood-Stage Vaccines for *Plasmodium falciparum* Malaria

Version: Version 7.0

Revision History: June 8, 2005; March 31, 2005; November 15, 2004; July 12, 2004, June 11, 2004

Volunteers: Healthy malaria-unexposed male and non-pregnant female volunteers 18 to 50 years of age

Number of Volunteers: 60 (6 groups of 10)

Trial Design: Open-label clinical trial

Immunization Schedule:

Group	Number of Volunteers	Immunization Schedule		
		Day 0	Day 28	Day 180
1	10	A	A	A
2	10	B	B	B
3	10	C	C	C
4	10	D	D	D
5	10	E	E	E
6	10	F	F	F
Total	60	A: 5 µg $MSP1_{42}$ -FVO/ Alhydrogel®<br>B: 20 µg $MSP1_{42}$ -FVO/ Alhydrogel®<br>C: 80 µg $MSP1_{42}$-FVO/ Alhydrogel®<br>D: 5 µg $MSP1_{42}$-3D7/ Alhydrogel®<br>E: 20 µg $MSP1_{42}$-3D7/ Alhydrogel®<br>F: 80 µg $MSP1_{42}$-3D7/ Alhydrogel®		

Product Description: The $MSP1_{42}$ vaccine formulations to be studied are from two different lines of *Plasmodium falciparum* (FVO and 3D7), each produced as recombinant proteins expressed in *Escherichia coli*. Bulk $MSP1_{42}$ antigens were purified from solubilized inclusion bodies and the correctly folded material purified by a combination of metal affinity, anion-exchange and size-exclusion chromatography. Purified $MSP1_{42}$-FVO and $MSP1_{42}$-3D7 drug substances were adsorbed individually onto aluminum hydroxide gel (Alhydrogel®, Brenntag, Denmark).

Time Period: A total of 64 weeks including vaccinations, study procedures, and follow-up of all volunteers. Each volunteer will be followed for a total of 52 weeks (maximum of 60 weeks including screening).

1.0 INTRODUCTION

1.1 Background

As reported by the World Health Organization in 2002, the worldwide incidence of malaria is approximately 300 million clinical cases annually, with approximately one million deaths per year attributed to malaria alone or in combination with other diseases [1]. Most of the malaria mortality occurs in sub-Saharan Africa and in children under 5 years of age. Of the four species of malaria parasite that infect humans, *Plasmodium falciparum* is responsible for the majority of these deaths. Mounting drug resistance of the malaria parasite, as well as widespread resistance of mosquitoes to insecticides, make these control strategies increasingly unrealistic. A vaccine that would reduce both mortality and morbidity secondary to *P. falciparum* infection would be a valuable resource in the fight against this disease.

P. falciparum has a complex life cycle. Sporozoites, the infectious stage of the parasite, are transmitted to humans through the saliva of infected female mosquitoes while taking a blood meal. Sporozoites travel through the bloodstream to the liver, where they invade hepatocytes and then undergo a series of divisions developing into merozoites. Six to 10 days after invasion, the hepatocytes rupture, releasing thousands of merozoites into the bloodstream. These merozoites invade erythrocytes, multiply and after 2 days, release progeny merozoites, which subsequently invade new erythrocytes to continue the asexual blood-stage cycle. Clinical symptoms in humans are due to this asexual blood-stage of the parasite's life cycle. A small percentage of merozoites do not multiply after invading erythrocytes, but instead differentiate into gametocytes. These gametocytes are ingested by a female mosquito during a subsequent blood meal and undergo sexual reproduction in the mosquito midgut, producing a zygote. The zygote matures and releases sporozoites that migrate to the mosquito's salivary glands, thus completing the life cycle.

Proteins expressed by *P. falciparum* are generally specific to one stage of the parasite's life cycle. Several *P. falciparum* merozoite antigens have been identified as potential blood-stage vaccine candidates [2]. Merozoite surface protein 1 (MSP1), the first protein to be identified on the surface of the blood-stage parasite [3], is synthesized as a ~200kDa polypeptide. MSP1 is processed at, or just prior to merozoite release from the red blood cell into four smaller fragments, $MSP1_{83}$, $MSP1_{30}$, $MSP1_{38}$ and $MSP1_{42}$ which form a non-covalently associated complex [4]. The 42kDa fragment $MSP1_{42}$is responsible for holding the complex together and tethering it to the surface of the merozoite via a glycosylphosphatidylinositol (GPI) anchor [5,6]. At the time of merozoite invasion of erythrocytes, $MSP1_{42}$ undergoes a secondary processing event and is cleaved into $MSP1_{33}$ and $MSP1_{19}$ [7]. $MSP1_{19}$ is the only part of MSP1 carried into the newly invaded erythrocyte on the surface of the parasite [8].

Several studies have suggested that the C-terminal region of MSP1 is the target of protective immune responses. Both monoclonal and polyclonal antibodies to $MSP1_{42}$ or $MSP1_{19}$ have been shown to inhibit erythrocyte invasion *in vitro* [9,10]. These antibodies when passively transferred into mice protected against blood-stage challenge with *Plasmodium yoelii* [29,30]. Additional studies in mice and monkeys immunized with parasite-derived MSP1 have demonstrated protective immunity in parasite challenge experiments [11,12]. Recombinant proteins of the C-terminal region, derived from *Escherichia coli*, *Saccharomyces cerevisiae,* baculovirus or transgenic milk, when used as vaccines also protect in mouse and monkey models

of malaria infection [13-22].

1.1.1 *MSP1$_{42}$ Vaccines*

Vaccines containing MSP1$_{42}$ proteins derived from two different lines of *P. falciparum,* FVO and 3D7, are being tested in this study. Both recombinant MSP1$_{42}$-FVO and MSP1$_{42}$-3D7 are highly purified 42,173 and 44,236 Da proteins respectively. Each were expressed by *E. coli* from synthetic genes corresponding to the published amino acid sequence for their respective *P. falciparum* lines (GenBank accession numbers: L20092 for FVO and AL929358 for 3D7). The recombinant MSP1$_{42}$-FVO and MSP1$_{42}$-3D7 drug substances were each purified from *E. coli* inclusion bodies under current Good Manufacturing Practice (cGMP) conditions. Following extensive quality control evaluation and toxicology testing the drug substances were formulated with Alhydrogel® to generate two drug products, MSP1$_{42}$-FVO/Alhydrogel® and MSP1$_{42}$-3D7/Alhydrogel® malaria vaccines.

1.2 Vaccine Description

1.2.1 *MSP1$_{42}$ Drug Substances*

Both recombinant MSP1$_{42}$-FVO and MSP1$_{42}$-3D7 are highly purified proteins that correspond to the external domain of MSP1$_{42}$ of *P. falciparum* FVO and 3D7 lines, respectively. The proteins consist of the MSP1$_{42}$ fragment sequence excluding the glycosylphosphatidylinositol (GPI) anchor sequence. The MSP1$_{42}$-FVO and MSP1$_{42}$-3D7 Drug Substances each contain an additional 8 amino acids not found in the native MSP1$_{42}$ sequence; the addition of a Leu-Glu dipeptide sequence results from gene cloning and a 6-histidine C-terminal tag to allow for efficient purification of the protein. As *E. coli* codon usage is significantly different from *P. falciparum*, the native MSP1$_{42}$ nucleotide sequences were optimized for expression in *E. coli*. The synthetic MSP1$_{42}$-FVO and MSP1$_{42}$-3D7 sequences were subcloned into the expression plasmids, pET-24d(+) and pET-24a(+) respectively (Novagen). The recombinant proteins MSP1$_{42}$-FVO and MSP1$_{42}$-3D7 were each expressed in *E. coli* and purified from solubilized inclusion bodies by a combination of metal affinity, anion-exchange and size-exclusion chromatography. The purification process was designed to separate full-length, correctly folded product from degraded material as well as nonproduct related impurities. MSP1$_{42}$-FVO and MSP1$_{42}$-3D7 bulk Drug Substances were supplied in sterile phosphate buffered saline (pH 7.4) containing 0.2% Polysorbate 80 (Tween 80), as a stabilizer. Both Drug Substances were manufactured under cGMP conditions at the Walter Reed Army Institute of Research (WRAIR), Bioproduction Facility (Silver Spring, Maryland). Prior to release, the drug substances underwent comprehensive quality control analysis to ensure purity, identity and integrity met specifications.

1.2.2 *Alhydrogel®*

Alhydrogel® (Brenntag, Denmark) is an aluminum hydroxide gel adjuvant. Aluminum hydroxide gels have been extensively used as an adjuvant in many licensed human vaccines. Aluminum-containing adjuvants are in routine human use and contained in many licensed human vaccines.

1.2.3 Vaccine Formulation, Drug Products

The final Drug Products were produced by the Pharmaceutical Development Section (PDS), Pharmacy Department, Clinical Center, National Institutes of Health (NIH), as single-dose vials of a cloudy suspension containing either $MSP1_{42}$-FVO or $MSP1_{42}$-3D7 adsorbed to Alhydrogel® (Brenntag Denmark) in sterile saline (0.9% sodium chloride) without additional stabilizers or preservatives. Three different formulations of each Drug Product, 10, 40 and 160 µg of protein/mL, were manufactured. Each formulation, containing 1,600 µg/mL Alhydrogel®, has been designed for delivery of a 0.5 mL dose and is supplied in a stoppered non-siliconized glass vial containing 0.7 mL of Drug Product. Prior to release, the Drug Product was subject to comprehensive quality control analysis to ensure purity, identity and potency.

1.3 Rationale

1.3.1 Preclinical Experience with $MSP1_{42}$-FVO and $MSP1_{42}$-3D7

The $MSP1_{42}$-FVO and $MSP1_{42}$-3D7 vaccines have been tested in mice, rabbits, and Aotus monkeys. A total of 5 individual preclinical animal studies have been conducted to assess the safety, immunogenicity and toxicity of the recombinant FVO and 3D7 $MSP1_{42}$ proteins when administered predominantly with Alhydrogel® (see **Appendix A** for a tabular summary of the preclinical trials) prior to human clinical trials. These studies have demonstrated the safety of $MSP1_{42}$-FVO and $MSP1_{42}$-3D7 when adjuvanted with Alhydrogel® and administered separately. Immunogenicity data from BALB/c mice, New Zealand White rabbits, and *Aotus nancyami* monkeys have demonstrated that each animal species developed significant anti- $MSP1_{42}$ antibody responses without clinically significant safety issues. Repeated intramuscular administration of the maximum human dose to rabbits did not exhibit any specific signs of systemic or local toxicity.

1.3.2 Clinical Experience with $MSP1_{42}$ Vaccines

The vaccine formulations in this protocol have not been tested in human trials. However, Walter Reed Army Institute of Research (WRAIR) has a candidate *P. falciparum* $MSP1_{42}$-3D7 malaria vaccine (FMP1) adjuvanted with GlaxoSmithKline Biologicals' ASO2A under Investigational New Drug (IND) application to the U.S. Food and Drug Administration (FDA). FMP1 has been tested in Phase 1 and Phase 1/2a human trials in both the United States and in Africa. To date, 2 trials in the United States have been done in healthy non-immune adult volunteers, 1 adult Phase 1 trial has been completed in Kenya, 1 adult Phase 1 trial is underway in Mali, and 1 pediatric Phase 1 trial (children aged 1 to 5 years) has recently been started in Kenya. No data is yet available for the pediatric trial recently started in Kenya.

In total, FMP1 adjuvanted with ASO2A has been administered, either alone or in combination with the investigational malaria vaccine RTS,S/ASO2A, to a total of 100 adults (a total of approximately 297 doses). No vaccine-related SAEs have occurred, and good immunogenicity to this antigen has been demonstrated. In the two trials conducted in healthy, malaria-unexposed adults in the United States, 20 volunteers received 89 doses of FMP1/ASO2A alone; mild to moderate injection site pain was the most common recorded adverse event, with one episode of severe injection site pain being the worst reaction observed.

The safety results from the two adult Phase 1 FMP1/ASO2A trials conducted in Africa are still blinded (volunteers were randomized to receive either the FMP1 or Imovax® Rabies vaccine). However, when considering the FMP1/ASO2A and Rabies vaccines together, 80 adults have received a total of 237 doses. Mild to moderate injection site pain and swelling were the most commonly observed adverse events, while mild to moderate arm motion limitation, fever, myalgia, malaise, arthralgia, nausea, and headache were also observed. In the Kenyan study, approximately a third of volunteers experienced severe pain at the site of injection, although this typically lasted only one or two days, and resolved without intervention. In the Malian study, approximately one quarter of volunteers experienced severe swelling at the injection site (defined as swelling greater than 50 mm in diameter), although this did not typically cause discomfort, and resolved within one to two days.

1.3.3 Clinical Experience with Aluminum-Based Adjuvants

Several licensed vaccines contain aluminum-based adjuvants, including the recombinant Hepatitis B vaccine (Recombivax HB®) and the diphtheria-tetanus toxoids vaccine (DT) [27, 28]. For these two aluminum-adsorbed vaccines, local reactions such as pain, tenderness and swelling were experienced in 7.6% to 16.7% of volunteers in studies that included over 1,200 healthy adults. Fever was seen in 3.2% to 9.3%, headache in 4.1% and other systemic symptoms such as fatigue, malaise, nausea and diarrhea at lower frequencies. Urticaria has been reported in 0.1% of individuals vaccinated with Recombivax HB®. These data are based in part on Recombivax HB® vaccine formulations that contained the preservative thimerosal, which may increase reactogenicity. Nonetheless, Recombivax HB® may be a particularly useful comparator vaccine, as it consists of a recombinant protein expressed in *S. cerevisiae* and is administered intramuscularly.

1.4 Clinical Development Plan

The first $MSP1_{42}$/Alhydrogel® Phase 1 trial will be carried out over 64 weeks in the United States. The study will be an open-label, dose-escalating Phase 1 clinical trial in healthy adult volunteers designed to evaluate the safety, reactogenicity and immunogenicity of the $MSP1_{42}$-FVO/Alhydrogel® and $MSP1_{42}$-3D7/Alhydrogel® vaccines in malaria-unexposed individuals. The maximum human dose of 80 µg/0.5 mL injected IM was tested in a rabbit toxicology study, and no clinically significant adverse effects were observed. Therefore, this dose was deemed to be reasonable.

Assuming no significant safety issues are identified in this initial Phase 1 trial, we plan to proceed with a Phase 1 trial of a combination vaccine of $MSP1_{42}$-FVO and $MSP1_{42}$-3D7 formulated on Alhydrogel® where the optimal dose ratio of each $MSP1_{42}$ serotype will be determined from the first Phase 1 trial as judged by safety and immunogenicity at Day 42. A Phase 1 trial of the combination vaccine formulation will be repeated in healthy, malaria-exposed adults in a malaria-endemic region, given the possibility that the safety of this vaccine formulation may be different in such a population. Provided no safety concerns become apparent, age de-escalation, Phase 2, and eventually Phase 3 clinical trials will be undertaken in malaria-endemic areas.

1.4.1 Participation of Children

The vaccine candidates being tested in this protocol have not yet been tested in humans. It is felt that insufficient data are available to judge the potential risk in children. Once safety is established in adults in the United States and then in an endemic region, we intend to age de-escalate to children in malaria endemic regions.

2.0 OBJECTIVES

2.1 Primary Objective

To determine the frequency and severity of vaccine-related AEs, for each dose.

2.2 Secondary Objectives

1. To determine the dose that generates the highest serum antibody levels to homologous $MSP1_{42}$ antigen at Day 42.
2. To assess and compare the duration of specific antibody response to homologous and heterologous $MSP1_{42}$ over a 12 month period.
3. To measure the effect of boosting at 6 months on specific antibody levels.

The following will be for information only:

1. To measure the ability of vaccine induced antibody to inhibit FVO and 3D7 *P. falciparum* growth in vitro.
2. To determine the relationship between anti- $MSP1_{42}$ antibody levels and degree of in vitro parasite growth inhibition

3.0 STUDY DESIGN

3.1 Overall Design

The study is an open-label Phase 1 dose-escalating clinical trial in healthy adult volunteers designed to evaluate the safety and immunogenicity of the $MSP1_{42}$-FVO and $MSP1_{42}$-3D7 malaria vaccines formulated on Alhydrogel®. Volunteers will be recruited and screened; those determined to be eligible, based on the inclusion and exclusion criteria described in **Section 4.0** in this protocol, will be enrolled in the study. After providing written informed consent, volunteers will undergo eligibility screening, including medical history, physical examination, hematology testing, liver and renal function testing, HIV, Hepatitis B and C screening and urinalysis. Urine pregnancy testing will be performed on female volunteers. Clinically significant abnormalities will be reviewed with volunteers and referral for follow-up care will be provided. For eligible participants, the Day 0 visit will be scheduled for receipt of the first dose of vaccine. Vaccinated volunteers will be observed for immediate reactions following each vaccination for 60 minutes. Volunteers will return to the clinic on Days 1, 3, 7 and 14 following each vaccination for clinical assessment. See **Table 1** for a tabular description of the vaccination schedule.

Sixty volunteers will be enrolled and assigned to one of six groups: 10 volunteers in each of three $MSP1_{42}$-FVO/Alhydrogel® groups will receive either 5 µg, 20 µg or 80 µg dose of

MSP1$_{42}$-FVO protein respectively, and 10 volunteers in each of three MSP1$_{42}$-3D7/Alhydrogel® groups will receive either 5 µg, 20 µg or 80 µg dose of MSP1$_{42}$-3D7 antigen as outlined in **Table 1**. All vaccines will contain the same amount of aluminum, 419 µg /dose. As with other aluminum-adsorbed vaccines, hypersensitivity reactions would be expected to occur within the first 24 hours after receipt of the vaccine, and other severe local or systemic reactions within 72 hours of vaccination. Prior to dose escalation, safety data up to and including Day 35 post-first vaccination and 7 days post-second vaccination will be available for the lower dose cohort for each individual product for review by the Safety Monitoring Committee (SMC). The trial will not proceed to the next dose cohort if, in the clinical judgment of the SMC, the next higher dose would pose an unacceptable safety risk to the volunteers. The MSP1$_{42}$-FVO/Alhydrogel® and MSP1$_{42}$-3D7/Alhydrogel® formulations will be considered individually by the SMC prior to dose escalation.

Table 1: MSP1$_{42}$/Alhydrogel® Vaccine[1] Dose Escalation Schedule

**Time** (Week)	**Group A:** (n = 10) 5 µg dose MSP1$_{42}$-FVO	**Group D:** (n = 10) 5 µg dose MSP1$_{42}$-3D7	**Group B:** (n = 10) 20 µg dose MSP1$_{42}$-FVO	**Group E:** (n = 10) 20 µg dose MSP1$_{42}$-3D7	**Group C:** (n = 10) 80 µg dose MSP1$_{42}$-FVO	**Group F:** (n = 10) 80 µg dose MSP1$_{42}$-3D7
0	Vaccination 1, Day 0 (n = 10)					
4	Vaccination 2, Day 28 (n = 10)					
6[2]			Vaccination 1, Day 0 (n = 10)			
10			Vaccination 2, Day 28 (n = 10)			
12[2]					Vaccination 1, Day 0 (n = 10)	
16					Vaccination 2, Day 28 (n = 10)	
24	Vaccination 3, Day 180 (n =10)					
30			Vaccination 3, Day 180 (n = 10)			
36					Vaccination 3, Day 180 (n = 10)	

[1] All vaccines contain the equivalent of 419 µg Aluminum per dose.
[2] Dose escalation will occur at a minimum of two weeks after the lower dose cohort has received the second vaccination

3.2 Sample Size and Estimated Duration of Study

Sixty volunteers will be enrolled. The day 42 safety and immune responses will be analyzed following the groups C and F day 42 bleed (week 18). This analysis will form the basis for the design of a Phase 1 trial to test a combination of MSP1$_{42}$ -FVO and MSP1$_{42}$-3D7 on Alhydrogel®. This trial is expected to last for a total of 64 weeks. Each volunteer will be followed for 52 weeks from the time of the first injection, for a maximum of 60 weeks in including screening.

3.3 Group Allocation

Volunteers will be assigned to one of six vaccine groups, defined by MSP1$_{42}$ serotype and dose. Volunteers will alternately be placed in either the FVO or 3D7 MSP1$_{42}$ group after enrollment into a dose cohort.

4.0 SELECTION AND ENROLLMENT OF VOLUNTEERS

4.1 Inclusion Criteria

1. Males or females between 18 and 50 years, inclusive.
2. Good general health as determined by means of the screening procedure.
3. Available for the duration of the trial (52 weeks).
4. Willingness to participate in the study as evidenced by signing the informed consent document.

4.2 Exclusion Criteria

1. Pregnancy as determined by a positive urine human chorionic gonadotrophin (ß-hCG), if female.
2. Participant unwilling to use reliable contraception methods for the duration of the trial.
3. Currently lactating and breast-feeding (if female).
4. Evidence of clinically significant neurologic, cardiac, pulmonary, hepatic, rheumatologic, autoimmune, or renal disease by history, physical examination, and/or laboratory studies including urinalysis.
5. Evidence of obesity; BMI must be < 35. Body mass index = ((weight in pounds)/(height in inches) x (height in inches)) x 703.
6. Behavioral, cognitive, or psychiatric disease that in the opinion of the investigator affects the ability of the volunteer to understand and cooperate with the study protocol.
7. Laboratory evidence of liver disease (aspartate aminotransferase [AST] and/or alanine aminotransferase [ALT] greater than 1.25 times the upper limit of normal of the testing laboratory).
8. Laboratory evidence of renal disease (serum creatinine greater than the upper limit of normal of the testing laboratory).
9. Laboratory evidence of hematologic disease (absolute neutrophil count < 1,500/mm^3; hemoglobin < 0.9 times the lower limit of normal of the testing laboratory, by sex; or platelet count < 140,000/mm^3).
10. Other condition that in the opinion of the investigator would jeopardize the safety or rights of a volunteer participating in the trial or would render the subject unable to comply with the protocol.
11. Participation in another investigational vaccine or drug trial within 30 days of starting this study, or while this study is ongoing.
12. Volunteer has abused alcohol or illicit drugs during the past 6 months, by history and/or positive urine drug screen (any detectable levels) on Study Day 0 or 180.
13. History of a severe allergic reaction or anaphylaxis.
14. Severe asthma (emergency room visit or hospitalization within the last 6 months).
15. Positive ELISA and confirmatory Western blot tests for HIV-1.
16. Positive ELISA and confirmatory immunoblot tests for HCV.
17. Positive HBsAg by ELISA.
18. Known immunodeficiency syndrome.
19. Use of corticosteroids (excluding topical or nasal) or immunosuppressive drugs within 30 days of starting this study or while the study is ongoing.
20. Receipt of a live vaccine within past 4 weeks or a killed vaccine within past 2 weeks prior to entry into the study.

21. History of a surgical splenectomy and/or abnormal splenic function.
22. Receipt of blood products within the past 6 months.
23. Previous receipt of an investigational malaria vaccine.
24. Receipt of antimalarial prophylaxis during the past 12 months.
25. Prior malaria infection.
26. Travel to a malaria-endemic country during the past 12 months or planned travel to a malaria-endemic country during the course of the study.
27. History of a known allergy to nickel.

4.3 Treatments That Could Potentially Interfere with Vaccine-Induced Immunity

The following criteria should be checked at each visit. If any become applicable during the study, the participant will be excluded from receiving further doses of the study vaccine and will not be included in the immunogenicity evaluations after the time of exclusion. The participant will, however, be encouraged to remain in the safety evaluation for doses already received.

1. Use of any investigational drug or investigational vaccine other than the study vaccine during the study period.
2. Administration of chronic (defined as more than 14 days) immunosuppressants or other immune-modifying drugs 6 months prior to vaccination. (Topical and nasal steroids are allowed.)
3. Administration of a licensed vaccine during the period starting from Day 14 to Day 42 and from Days 166 to 194 (14 days before and after each vaccination).
4. Administration of immunoglobulins and/or any blood products up to 30 days after the last dose of vaccine.

4.4 Contraindications to Vaccination

The following criteria should be checked prior to each immunization and are contraindications to further immunization. However, the participant will be encouraged to remain in the safety evaluation for doses already received. Should a female volunteer become pregnant during the course of the study, she will be referred to an appropriate specialist and followed for the duration of the pregnancy.

1. Hypersensitivity reaction following administration of the study vaccine.
2. Pregnancy, as determined by a positive urine ß-hCG.

4.5 Indications for Deferral of Vaccination

The following AEs constitute grounds for deferral of vaccine administration at that point in time; if any one of these AEs occurs at the time scheduled for vaccination, the participant may be vaccinated at a later date, within the allowable time interval specified in the protocol, or withdrawn at the discretion of the investigator. The participant must be followed until resolution of the event as with any AE. If the participant is withdrawn from the study, he/she will be encouraged to remain in the safety evaluation for the duration of the study.

1. Oral temperature > 37.5°C at the time of vaccination will warrant deferral of immunization until fever and symptoms resolve.

2. Any other condition that in the opinion of the investigator poses a threat to the individual if immunized or that may complicate interpretation of the safety of the vaccine following immunization.

Such individual(s) will be followed in the clinic until the symptoms resolve or the window for immunization expires. No further vaccination will be performed if the participant does not recover (oral temperature ≤ 37.5°C and/or lack of symptoms) within the vaccination window described in Section 6.4 of this protocol. The participant, however, will be followed for safety and immunogenicity. If the individual meets any of the above criteria for deferral on the day of first immunization, the investigator may elect to exclude the participant from further participation in the study. Eligible alternates will then be vaccinated instead.

4.6 Subject Withdrawal Criteria

A volunteer will not be considered to have completed the trial if any of the following reasons apply. However, any volunteer who has received at least one dose of vaccine will be encouraged to remain in the safety evaluation for the duration of the study.

1. *Research terminated by sponsor or investigator* – applies to the situation where the entire study is terminated by the sponsor, or investigator for any reason.
2. *Withdrawal of consent* – applies to a subject who withdraws consent to participate in the study for any reason.
3. *Noncompliant with protocol* – applies to a volunteer who does not comply with protocol-specific visits or evaluations, on a consistent basis, such that adequate follow-up is not possible and the volunteer's safety would be compromised by continuing in the trial. Additionally, this applies to a volunteer who is lost to follow-up and is not reachable by telephone or other means of communication, and therefore not able to be located. In the event that a volunteer becomes incarcerated during the course of this study, every attempt will be made to continue to monitor for safety for the duration of the study.
4. *Other* – is used when previous categories do not apply and a written explanation is required.

5.0 VACCINE PREPARATION

5.1 Supplies

Research products for this protocol will be supplied to the study site pharmacist by the Pharmaceutical Development Section (PDS), Pharmacy Department, Clinical Center, National Institutes of Health (NIH), where the vaccines were formulated and vialed. Vaccines will be transported at 2°C to 8°C from the PDS at the NIH to the study site pharmacy with a temperature recording device. Upon receipt of vaccine supplies, the study site pharmacist will ensure that the temperature was maintained between 2°C to 8°C using the temperature recording device and it's associated computer program. The study site pharmacy will be responsible for maintaining the appropriate supply of vaccine and will request additional vaccine in writing through the IND sponsor.

$MSP1_{42}$/Alhydrogel® malaria vaccines are supplied as a cloudy suspension in single-dose vials. Each 2.0 mL vial contains 0.7 mL, of which 0.5 mL is the intended volume to be injected. 0.5 mL of vaccine contains the equivalent of 419 µg of aluminum as Alhydrogel® (The U.S. FDA upper allowable limit of aluminum adjuvants for injection in humans is 0.85-1.25 mg [31]) onto

which either 5 μg, 20 μg or 80 μg of recombinant $MSP1_{42}$-3D7 or $MSP1_{42}$-FVO have been bound. The Drug Product conforms to established requirements for sterility, safety and identity.

5.2 Vaccine Storage

$MSP1_{42}$/Alhydrogel® malaria vaccines should be maintained at 2°C to 8°C until just prior to administration. Vaccine should NOT be frozen at any time. Single-dose vials should be stored in the upright position.

5.3 Vaccine Accountability

The trial site pharmacists are responsible for maintaining an accurate inventory and accountability record of vaccine supplies for this study. Partially used vials may not be administered to other volunteers.

5.4 Disposition of Used/Unused Supplies

After administration of a vaccine dose, the single-dose vial will be returned to the trial site pharmacy, and vials will be accounted for and stored until monitoring by the study sponsor. Used vials may be disposed of according to study site protocol after monitoring has been completed. Final disposition of unused vaccine supplies will be determined by the Malaria Vaccine Development Branch, NIH, in conjunction with the IND Sponsor.

6.0 STUDY PROCEDURES

The following sections provide a detailed listing of the procedures and studies to be performed in this protocol at designated time points. The total volume of blood, approximately 330 mL (up to 380 mL for 80 μg groups who agree to additional blood volume being drawn on day 194), to be drawn over the duration of the trial is less than volume collected when donating a single unit of blood and should not compromise the health of trial participants.

6.1 Screening (Up to 60 Days Prior to Vaccination)

1. Explain the study and Informed Consent to the volunteer.
2. Ensure the subject has signed the Informed Consent and received a signed copy of the Informed Consent.
3. Pre-test counseling for HIV testing and ensure the subject has signed the HIV testing Informed Consent. Post-test counseling to follow when results obtained.
4. Elicit a complete medical history, including menstrual and contraceptive history and/or history of surgical sterility for female subjects.
5. Administer a complete physical examination.
6. Obtain approximately 20 mL of blood for hematology, biochemistry, serologic tests for viral hepatitis and HIV in all volunteers.
7. Obtain urine for urine dipstick testing, as well as urine β-hCG testing in females.
8. Counsel females to avoid becoming pregnant during the study.

6.2 Enrollment

Volunteers will respond to advertisements distributed by the clinical trial site (See **Appendix B and C**). After an initial phone screen by clinical trial staff, consisting of background information of the trial, a screening visit will be scheduled (See **Appendix D**). During this initial screening visit, the volunteer will read the consent form and be encouraged to ask questions. The volunteer may either sign the consent form during the screening visit or return after further consideration. Rolling recruitment and enrollment will occur to fill the low dose cohort (groups A & D) prior to the medium dose cohort (groups B & E) followed by the high dose cohort (groups C & F). After enrollment in to a dose group, volunteers will alternately be placed in either the $MSP1_{42}$-FVO/Alhydrogel® or $MSP1_{42}$-3D7/Alhydrogel® group.

6.3 Immunization Procedure

Volunteers will receive three immunizations, on Days 0, 28 and 180. Vaccine will be kept refrigerated at 2^oC to 8^oC until just before use. 0.5 mL will be delivered by IM injection in the deltoid muscle with a 21-gauge needle of appropriate length after preparation of the site with alcohol. Successive vaccinations will be given in alternating arms.

6.4 Clinical Monitoring and Evaluation

See **Appendix E** for a tabular representation of study procedures.

<u>Study Day 0 (Day of First Vaccination)</u>

1. Verify that Informed Consent was obtained.
2. Verify that all applicable eligibility criteria have been met.
3. Perform abbreviated history and physical exam, focusing on any acute complaints.
4. Obtain approximately 40 mL of blood for hematology, biochemistry, anti-$MSP1_{42}$ antibody ELISA and GIA.
5. For females, obtain a urine sample for β-hCG testing. Ensure that test is negative before vaccinating; a positive test will exclude the volunteer from the trial.
6. Obtain a urine sample for drug screening. Ensure that test is negative before vaccinating; a positive test will exclude the volunteer from the trial.
7. Record vital signs (blood pressure, temperature, heart rate, and respiratory rate).
8. Ensure CBC, AST/ALT and Creatinine are within protocol-defined limits (Section 4.0 Exclusion Criteria) before vaccinating; abnormal lab results may exclude the volunteer from the trial.
9. Administer the vaccine.
10. Observe for 60 minutes after vaccination to evaluate for immediate adverse reactions.
11. Education by study staff during 60-minute post-immunization wait period describing proper use of digital thermometers, injection-site reaction measurement and malaria vaccine side-effect diaries. Study staff will also discuss signs and symptoms of potential AEs.

<u>Study Day 1</u>

1. Perform basic history and physical exam (including injection site), emphasizing examination of any acute complaints.
2. Record vital signs.

Study Day 3

1. Perform basic history and physical exam (including injection site), emphasizing examination of any acute complaints.
2. Record vital signs.
3. Obtain approximately 10 mL of blood for hematology and biochemistry tests.

Study Day 7 +/- 1

1. Perform basic history and physical exam (including injection site), emphasizing examination of any acute complaints.
2. Record vital signs.
3. Collect Days 0-6 diary card.

Study Day 14 +/- 2

1. Perform basic history and physical exam (including injection site), emphasizing examination of any acute complaints.
2. Record vital signs.
3. Obtain approximately 20 mL of blood for hematology biochemistry, and anti-$MSP1_{42}$ antibody ELISA.

Study Day 28 +/- 4 (Day of Second Vaccination)

1. Perform basic history and physical exam (including injection site), emphasizing examination of any acute complaints.
2. Obtain approximately 20 mL of blood for hematology, biochemistry and anti-$MSP1_{42}$ antibody ELISA. Results will not be reviewed prior to vaccination.
3. For females, obtain a urine sample for β-hCG testing. Ensure that test is negative before vaccinating; a positive test will exclude the volunteer from the trial.
4. Record vital signs (blood pressure, temperature, heart rate and respiratory rate).
5. Administer the vaccine.
6. Observe for 60 minutes after vaccination to evaluate for immediate adverse reactions.
7. Education by study staff during 60-minute post-immunization wait period describing proper use of digital thermometers, injection-site reaction measurement tools and patient diaries. Study staff will also discuss signs and symptoms of potential AEs.

Study Day 29 (1 day after Second Vaccination)

1. Perform basic history and physical exam (including injection site), emphasizing examination of any acute complaints.
2. Record vital signs.

Study Day 31 (3 days after Second Vaccination)

1. Perform basic history and physical exam (including injection site), emphasizing examination of any acute complaints.
2. Record vital signs.
3. Obtain approximately 10 mL of blood for hematology and biochemistry tests.

Study Day 35 +/- 1 (7 days after Second Vaccination)

1. Perform basic history and physical exam (including injection site), emphasizing examination of any acute complaints.
2. Record vital signs.
3. Collect Days 0-6 diary card.

Study Day 42 +/- 2 (14 days after Second Vaccination)
1. Perform basic history and physical exam (including injection site), emphasizing examination of any acute complaints.
2. Record vital signs.
3. Obtain approximately 40 mL of blood for hematology, biochemistry, anti-$MSP1_{42}$ antibody ELISA and GIA.

Study Day 120 +/-14 (4 months after Second Vaccination)
1. Obtain approximately 30 mL of blood for anti-$MSP1_{42}$ antibody ELISA and GIA.

Study Day 180 +/- 14 (Day of Third Vaccination)
1. Perform basic history and physical exam (including injection site), emphasizing examination of any acute complaints.
2. Obtain approximately 40 mL of blood for hematology, biochemistry and anti-$MSP1_{42}$ antibody ELISA and GIA.
3. For females, obtain a urine sample for β-hCG testing. Ensure that test is negative before vaccinating; a positive test will exclude the volunteer from the trial.
4. Obtain a urine sample for drug screening. Ensure that test is negative before vaccinating; a positive test will exclude the volunteer from the trial.
5. Record vital signs (blood pressure, temperature, heart rate, and respiratory rate).
6. Ensure CBC, AST/ALT and Creatinine are within protocol-defined limits (Section 4.0 Exclusion Criteria) before vaccinating; abnormal lab results may exclude the volunteer from the trial.
7. Administer the vaccine.
8. Observe for 60 minutes after vaccination to evaluate for immediate adverse reactions.
9. Education by study staff during 60-minute post-immunization wait period describing proper use of digital thermometers, injection-site reaction measurement tools and patient diaries. Study staff will also discuss signs and symptoms of potential AEs.

Study Day 181 (1 day after Third Vaccination)
1. Perform basic history and physical exam (including injection site), emphasizing examination of any acute complaints.
2. Record vital signs.

Study Day 183 (3 days after Third Vaccination)
1. Perform basic history and physical exam (including injection site), emphasizing examination of any acute complaints.
2. Record vital signs.
3. Obtain approximately 10 mL of blood for hematology and biochemistry tests.

Study Day 187 +/- 1 (7 days after Third Vaccination)

1. Perform basic history and physical exam (including injection site), emphasizing examination of any acute complaints.
2. Record vital signs.
3. Collect Days 0-6 diary card.

Study Day 194 +/- 2 (14 days after Third Vaccination)
1. Perform basic history and physical exam (including injection site), emphasizing examination of any acute complaints.
2. Record vital signs.
3. Obtain approximately 40 mL of blood for hematology, biochemistry anti-$MSP1_{42}$ antibody ELISA and GIA.

Study Day 270 +/- 14 (90 days after Third Vaccination)
1. Perform basic history and physical exam, emphasizing examination of any complaints.
2. Obtain approximately 10 mL of blood for anti-$MSP1_{42}$ antibody ELISA.

Study Day 364 +/- 30 (180 days after Third Vaccination)
1. Perform basic history and physical exam, emphasizing examination of any complaints.
2. Record vital signs.
3. Obtain approximately 40 mL of blood for hematology, biochemistry, and anti-$MSP1_{42}$ antibody ELISA and GIA.

Volunteers in the 80 μg dose groups (Groups C & F) will be contacted at their day 180 visit to ask if they would be willing to have an additional, optional blood draw of 50 mL at their scheduled day 194 visit (14 days after the third vaccination). The total blood volume to be drawn on day 194, for those volunteers who agree, would therefore be 90 mL (scheduled blood draw is 40 mL). The total blood volume over the course of the study would then be increased to 380 mL. These serum samples would be used to create a high titer reference standard reagent to be used in future $MSP1_{42}$ vaccine trials. Serum samples of persons vaccinated with $MSP1_{42}$ would be compared against this reference standard for better comparison of vaccine immunogenicity between trials. The testing of vaccine efficacy in this and future trials of $MSP1_{42}$ will rely on the preparation of a well-defined reference standard reagent against which the sera from vaccinate volunteers can be tested and compared. An accepted approach for making such a reference reagent is to make a pool of sera with known anti-$MSP1_{42}$ antibody levels which is then used as a reference serum in ELISA assays. Such a reference serum would ideally be prepared in a large enough volume to be stored and used for several years. This reference serum will be used for research purposes only and will not have any commercial use or value.

6.5 Volunteer Symptom Diary

Volunteers will be asked to keep daily symptom diaries recording oral temperature as well as pain/tenderness, redness, induration at the injection site and any systemic signs or symptoms for 6 days following each immunization. The size of any injection-site reaction will be measured using a standardized clear plastic measurement device and recorded in the volunteer symptom diary.

6.6 Laboratory Testing

Using standard techniques, the clinical laboratory will perform the following tests:

1. Complete blood count plus white blood cell differential
2. Serum creatinine
3. AST
4. ALT
5. HIV assay (3rd generation ELISA with Western blot confirmation)
6. HBsAg ELISA
7. HCV (3rd generation ELISA with immunoblot confirmation)
8. Urinalysis (in the event of an abnormal urine dipstick test)
9. Urine drug screen (for amphetamines, barbiturates, benzodiazepines, cocaine, marijuana, opiates, PCP, propoxyphene, and alcohol)

Urine β-hCG testing will be performed at the clinical trial site using an U.S. Food and Drug Administration (FDA)-approved urine pregnancy test kit. Urine dipstick testing will be performed at the trial site using an FDA-approved product. Urine drug screen testing will be performed at the clinical laboratory using EIA.

The anti-$MSP1_{42}$ antibody levels and growth inhibition assays will be performed at the MVDB in Rockville, Maryland. Frozen sera will be shipped from the trial site to the MVDB on dry ice in batches at the discretion of the trial site with consultation from the MVDB. An inventory of each shipment will be maintained by both the trial site and the MVDB.

6.7 Immunologic Testing

6.7.1 Antibody Assay (ELISA)

Serum antibody levels to the $MSP1_{42}$ antigens will be measured by ELISA. Assays will be performed for both 3D7 and FVO $MSP1_{42}$ proteins. Briefly, microwell plates (Dynex Technologies) are coated overnight at 4°C with 100 µL/well of antigen solution (1 µg/mL). Plates are washed with TRIS-buffered saline (TBS) containing 0.1% Tween-20 (0.1% T-TBS) and blocked with TBS containing 5% skim milk powder for 2 hours at room temperature. After washing with 0.1% T-TBS, serum samples at a dilution of 1:5,000 in 0.5% T-TBS containing 5% skim milk powder are added in triplicate and incubated for 2 hours at room temperature. After incubation, unbound antibodies are removed by washing the plates with 0.1% T-TBS, and 100 µL of alkaline phosphatase-conjugated goat anti-human IgG solution (Kirkegaard & Perry Labs, Gaithersburg, Maryland, 1:1,000 dilution in 0.5% T-TBS containing 5% skim milk powder,) is added to each well and incubated for 2 hours at room temperature. Plates are then washed with 0.1% T-TBS, followed by adding 100 µL of phosphatase solution (Sigma, St. Louis, Missouri) to each well; the plates are then covered with aluminum foil and incubated for 20 minutes at room temperature for color development. The plates are read immediately at 405 nm with a microplate reader (Spectramax 340PC Molecular Devices). The optical density values are used to determine anti-$MSP1_{42}$ antibody concentration by comparison to a standard curve generated with known positive control sera included on each ELISA plate.

6.7.2 Growth Inhibition Assay (GIA)

The GIA is designed to determine whether anti-$MSP1_{42}$ antibodies obtained from an immunized animal or person can inhibit the process of merozoite invasion into red cells. In this assay, synchronized blood-stage parasites are incubated with sera from volunteers for a period of 40 hours in vitro. During this period, merozoites emerge from the infected red cells, invade normal red cells and initiate a new growth cycle. Parasite growth and development in the newly invaded red cells are assessed by quantitating the activity of a parasite metabolic enzyme, lactate dehydrogenase. Enzyme activity determined by a colorimetric assay is proportional to the number of parasites. GIA results with immune sera are compared to results with normal nonimmune sera and then expressed as percent inhibition of parasites.

7.0 ADVERSE EVENTS MONITORING AND REPORTING

7.1 Definitions

7.1.1 Adverse Event (AE)

An AE is any untoward medical occurrence in a trial participant administered the experimental vaccine and that does not necessarily have a causal relationship with vaccination. An AE can therefore be any unfavorable and unintended sign (including an abnormal laboratory finding), symptom, or disease temporally associated with the use of the investigational vaccine, whether or not related to it. This includes an exacerbation of pre-existing conditions and intercurrent illnesses. Unchanged pre-existing conditions will not be included as an adverse event. All AEs must be graded for intensity and relationship to the investigational vaccine as described in **Sections 7.2.2** and **7.2.3** in this protocol.

7.1.2 Serious Adverse Event (SAE)

An SAE is an AE, whether considered related to the investigational vaccine or not, meeting one of the following conditions:

1. Death during the period of protocol-defined surveillance
2. Life threatening: defined as an event that places a subject at immediate risk of death at the time of the event and does not refer to an event that hypothetically might have caused death were it more severe
3. Hospitalization during the period of protocol-defined surveillance: defined as at least an overnight stay in the hospital or emergency ward for treatment that would have been inappropriate if administered in the outpatient setting
4. Results in a congenital anomaly or birth defect
5. Results in a persistent or significant disability or incapacity: defined as a substantial disruption of the study participant's ability to carry out normal life functions
6. Any other important medical event that may not result in death, be life threatening, or require hospitalization, may be considered a serious AE when, based upon appropriate medical judgment, the event may jeopardize the subject and may require medical or surgical intervention to prevent one of the outcomes listed above

7.2 Assessment of Adverse Events

7.2.1 Identification of AEs

Assessment of safety will include clinical observation and monitoring of hematological, chemical and immunologic parameters. Safety will be evaluated by monitoring of volunteers for local and systemic adverse reactions during the course of the trial. Volunteers will be closely observed in the clinic for 60 minutes following each immunization. Additionally, volunteers will return to the clinic on Days 1, 3, 7 and 14 following each vaccination for clinical assessments. For 6 days after each immunization, volunteers will be asked to keep daily diaries of symptom recording oral temperature, as well as a subjective assessment of the extent of induration, erythema, pain/tenderness at the site of injection, and any systemic signs and symptoms. The size of any injection-site reaction will be measured using a standardized clear plastic measurement device and recorded in the volunteer symptom diary. All AEs will be graded for intensity and relationship to study product as described in **Section 7.2.2** in this protocol. A study clinician will be available by telephone or pager 24 hours a day during the study evaluation period. Should a volunteer call a study clinician to report an adverse event, it will be determined at that time if an extra visit(s) will be scheduled, and/or appropriate medical advice will be provided. Additionally, all calls will be documented in the volunteer's study chart, and discussed with the Principal Investigator.

7.2.2 Determination of Severity

All AEs will be assessed by the investigator using the following protocol-defined grading system:

Grade 0 (None)

Grade 1 (Mild): No effect on activities of daily living

Grade 2 (Moderate): Partial limitation in activities of daily living (can complete ≥ 50% of baseline), or treatment given

Grade 3 (Severe): Activities of daily living limited to < 50% of baseline, or medical evaluation required

Intensity of the following AEs will be assessed by the investigator as described in **Table 2**. All laboratory AEs will be graded in severity following the toxicity table in **Appendix G**. Unexpected adverse events not described in the protocol will be graded according to the common toxicity table in **Appendix H**.

Table 2: Assessment of Adverse Event Intensity

AdverseEvent	Grade	Intensity
Pain at injection site	0	Absent
	1	Pain that is easily tolerated
	2	Pain that interferes with daily activity
	3	Pain that prevents daily activity

Erythema at injection site	0<br>1<br>2<br>3	0 mm<br>>0 - ≤20 mm<br>>20 - ≤50 mm<br>>50 mm
Swelling at injection site	0<br>1<br>2<br>3	0 mm<br>>0 - ≤20 mm<br>>20 - ≤50 mm<br>>50 mm
Fever (oral)	0<br>1<br>2<br>3	≤99.5°F (≤37.5°C)<br>>99.5°F - 100.4°F (>37.5°C – 38°C)<br>>100.4°F - 102.2°F (>38°C – 39°C)<br>>102.2°F (>39°C)
Headache	0<br>1<br>2<br>3	None<br>Headache that is easily tolerated<br>Headache that interferes with daily activity<br>Headache that prevents daily activity
Nausea	0<br>1<br>2<br>3	None<br>Nausea that is easily tolerated<br>Nausea that interferes with daily activity<br>Nausea that prevents daily activity
Malaise	0<br>1<br>2<br>3	None<br>Malaise that is easily tolerated<br>Malaise that interferes with daily activity<br>Malaise that prevents daily activity
Myalgia	0<br>1<br>2<br>3	None<br>Myalgia that is easily tolerated<br>Myalgia that interferes with daily activity<br>Myalgia that prevents daily activity
Arthralgia	0<br>1<br>2<br>3	None<br>Joint pain that is easily tolerated<br>Joint pain that interferes with daily activity<br>Joint pain that prevents daily activity
Urticaria	0<br>1<br>2<br>3	None<br>Requiring no medications<br>Requiring PO or topical treatment or IV medication or steroids for ≤24 hours<br>Requiring IV medication or steroids for >24 hours

7.2.3 Association with Receipt of the Study Vaccine

All AEs will have their possible relationship to study vaccine assessed using the following terms:

Definite: Clear-cut temporal association, and no other possible cause.

Probable: Clear-cut temporal association and a potential alternative etiology is not apparent.

Possible: Less clear temporal association; other etiologies also possible.

Remote: Temporal association between the AE and the vaccine or the nature of the event is such that the vaccine is not likely to have had any reasonable association with the observed illness/event (cause and effect relationship improbable but not impossible).

Not Related: The AE is completely independent of vaccine administration; and/or evidence exists that the event is definitely related to another etiology.

The degree of certainty with which an AE can be attributed to administration of the study vaccine will be determined by how well the event can be understood in terms of one or more of the following:

1. The event being temporally related with vaccination or reproduced on re-vaccination.
2. A reaction of similar nature having previously been observed with this type of vaccine and/or formulation.
3. The event having often been reported in the literature for similar types of vaccines.

All local (injection-site) reactions will be considered causally related to vaccination.

7.3 Adverse Event Reporting

All SAEs will be reviewed by a study physician, recorded on the appropriate SAE form, and followed through to resolution by a study physician. All SAEs will be reported by telephone or fax within 1 working day of notification of the SAE occurrence to all of the following:

- IND Sponsor, Regulatory Compliance and Human Subjects Protection Branch Safety Section (RCHSPB Safety)/NIAID: Phone: 301-846-5301, Fax: 301-846-6224; email: RCHSPSafety@mail.nih.gov.
- Heartland Institutional Review Board (Clinical Trial Site IRB): Phone: 913-752-8607, Fax: 913-752-8652

Following notification from the investigator, RCHSPB as the IND sponsor, will report events that are both serious and unexpected that are possibly, probably or definitely related to the vaccine, to the FDA within the required timelines: fatal and life-threatening events within 7 calendar days (by phone or fax) and all other SAEs in writing within 15 calendar days. In addition, the Principal Investigator will notify the MVDB within 1 working day of notification of the SAE occurrence, who will in turn notify the NIAID IRB and MVI/PATH. MVI will then notify the PATH Human Subjects Protection Committee (HSPC). All SAEs not listed as possibly, probably or definitely related will be reported to the FDA at least annually in a summary format and to the IRBs as required by the institution. All local and systemic reactions not meeting the criteria for "serious adverse events" will be captured on the appropriate case report form (CRF). These events will be followed to resolution.

7.4 Adverse Event Monitoring

7.4.1 Local Safety Monitor

An independent local safety monitor, Bruce Short, MD, has been appointed for oversight of safety in this trial. The local safety monitor will be available to advise the investigators on trial-related medical questions or problems, and act as a representative for the volunteers' welfare. Additionally, the local safety monitor may ask to convene a safety monitoring committee meeting for review of any safety issue or adverse event.

7.4.2 Safety Monitoring Committee

MVDB in consultation with MVI/PATH will select three independent monitors to advise RCHSPB and the study investigators on the trial. These individuals will be independent of MVDB, the clinical trial site and MVI/PATH. The SMC's primary responsibility will be to

monitor participant safety. The Principal Investigator is responsible for ensuring that the SMC is aware of all new safety information. The SMC will periodically review individual and cumulative participant data on safety and enrollment when making recommendations regarding the safe continuation of the study. If no stopping criteria are met (**Section 7.5**), dose escalation will proceed with approval from the SMC. The SMC will review cumulative safety data for evidence of study-related AEs, adherence to the protocol, and factors that may affect outcome or study data such as protocol violations and losses to follow-up. The SMC will meet 7 to 14 days following the second immunization of the 5 µg and 20 µg dose groups to approve dose escalation, and whenever the need should arise, to review study conduct and cumulative safety data, as well as at the discretion of the SMC and/or study investigators.

7.5 Stopping Criteria

If a dose of vaccine is considered unacceptably reactogenic, dose escalation and/or additional vaccinations will be suspended until reviewed with the SMC and study sponsor (RCHSPB). The communications from the SMC will subsequently be forwarded by the investigators to the NIAID IRB, the PATH HSPC and the clinical trial site IRB. All local adverse events will be routinely reviewed by the SMC prior to granting approval for dose escalation.

The following criteria will be used to define unacceptable reactogenicity of either of the $MSP1_{42}$ malaria vaccines:

1. One or more volunteers experience a SAE (as defined in **Section 7.1.2** in this protocol) that is determined to be possibly, probably or definitely related to the vaccine (as defined in **Section 7.2.3** in this protocol), **OR**
2. One or more volunteers experience a hypersensitivity reaction (as defined in Appendix H) that is probably or definitely related to the vaccine, **OR**
3. Any severe clinical illness occurs that is not explained by a diagnosis that is unrelated to vaccination, **OR**
4. One or more volunteers in a single-dose cohort experience a Grade 3 or higher laboratory abnormality, or Grade 3 systemic AE that is determined to be possibly, probably or definitely related to the vaccine, as defined in **Section 7.2.2** in this protocol.

8.0 DATA COLLECTION AND MONITORING

8.1 Source Documentation

Complete source documentation (laboratory test reports, hospital or medical records, etc.) is required for every study subject for the entire duration of the study. Clinical Report Forms (CRFs) and volunteer symptom diaries will be used to record data for subjects enrolled in the study. The Investigator is responsible for the accuracy and completeness of the data reported to the IND Sponsor in the CRFs and diaries. Data reported in the CRFs derived from source documents should be consistent with source documents or the discrepancies should be explained.

8.2 Study Documentation

Study-related documentation will be completed as required by the IRBs, the IND Sponsor and regulatory authorities. Continuing review documentation will be submitted by the Investigator to

the IRBs on the anniversary date of initial review as specified by each IRB. An annual report will be submitted by the RCHSPB to the FDA on the anniversary date that the IND for $MSP1_{42}$/Alhydrogel® malaria vaccine went into effect. These reports will provide a brief description of the progress of the investigation as outlined in 21 *Code of Federal Regulations* 312.33 and will include any revisions of the protocol.

The Investigator will maintain adequate records of the disposition of the investigational product, including dates, quantity and use by subjects. If the study is terminated, suspended or completed, final disposition of unused vaccine supplies will be determined by the Malaria Vaccine Development Branch, in conjunction with the IND Sponsor.

In addition to the study-related documentation required by the regulatory authorities, the MVDB will also submit two reports to the IND Sponsor and Funding Sponsor (MVI/PATH). The first, or interim, report will be completed after the serologic data from the 6-week blood draw has been compiled. This interim report, based on the serologic response that is observed 2 weeks after the second immunization, will serve as the basis for deciding whether to continue with future Phase 1 testing of the formulation. A final report will be submitted by the MVDB to the IND Sponsor and Funding Sponsor after trial completion.

8.3 Retention of Records

Trial-related documents will be maintained by the Investigator for a period of 2 years after final marketing approval of the vaccine, or for 2 years following the formal discontinuation of clinical development of the product. The IND Sponsor is required to inform the Investigator as to when such documents need no longer be retained. Storage of all trial-related documents will be such that confidentiality will be strictly maintained.

8.4 Protocol Revisions

No revisions to this protocol will be permitted without documented approval from both the IND Sponsor and the IRBs that granted the original approval for the study. This does not apply to changes made to reduce discomfort or avert risk to study volunteers. Furthermore, in the event of a medical emergency, the Investigator shall perform any medical procedures that are deemed medically appropriate. The Investigator must notify the IND Sponsor of all such occurrences. Any change to the protocol will be submitted to the participating IRBs as a protocol amendment and changes not affecting risk to volunteers may be expedited, as appropriate.

8.5 Clinical Investigator’s Brochure

Investigators will receive the current version of the Clinical Investigator’s Brochure, which comprehensively describes all the available preclinical experience with the experimental vaccine. If relevant new information becomes available during the course of the trial, the Investigators will receive a revised Investigator’s Brochure or an amendment to the current version.

8.6 Study Monitoring

The IND Sponsor will monitor through delegated responsibility, all aspects of the study, with respect to current Good Clinical Practices (GCP), for compliance with applicable government regulations. Prior to the start of the study, the Investigator will be informed of the frequency of monitoring visits and will be given reasonable notification prior to each visit. The objectives of

a monitoring visit will be to verify the prompt reporting of SAEs, to check the availability of the signed Informed Consent, GCP adherence to the protocol, and to compare CRFs and spreadsheets with source data for completeness and accuracy. During the monitoring visit, the Investigator (and/or designee) and other study personnel should be available to discuss the study. Study documents must be available for review throughout the course of the study. The IND Sponsor will retain originals of the FDA Form 1572 and copies of other study documents as deemed necessary.

9.0 STATISTICAL CONSIDERATIONS

9.1 General Design

The goal of this Phase 1 vaccine trial is to demonstrate safety and immunogenicity of $MSP1_{42}$/Alhydrogel® malaria vaccines in human volunteers. The results from this trial will be used to inform dosing and scheduling in subsequent trials.

9.1.1 Description of the Statistical Methods to Be Employed

The purpose of this trial is to estimate event rates and patterns of immune responses as well as to compare these rates and patterns in different doses of the study vaccines and to compare the response by the two $MSP1_{42}$ vaccine serotypes. This section briefly describes the statistical methods to be used. A detailed analytic plan will fully describe the methods. The analytic plan will discuss the planned approaches to missing data. Listings will show all observed data and, if applicable, impute values and the approaches taken for imputation.

Estimates will be presented with their 95% confidence intervals. Descriptive approaches will be used to meet the protocol objectives as stated in **Section 2.0** of this protocol. In particular, the immunologic response and the adverse events for each of the 60 volunteers will be presented as individual graphs. Formal statistical tests, as outlined below, will be used to compare doses and to compare $MSP1_{42}$ serotypes. Results will be presented in tabular format, as well as graphically where appropriate.

Because of the small sample size of this study, statistical tests will be performed without correction for multiplicity. A nominal Type I error rate of 10 percent will be used. Most of the analyses of immunogenicity will be based on a longitudinal mixed model with terms for dose group and $MSP1_{42}$ serotype.

<u>Primary Objective</u>: To determine the frequency and severity of vaccine-related AEs for each dose.

a. Summarize the frequency of immediate, systemic and local AEs.
b. Line listing of individual clinical and laboratory AEs as classified by immediate (within the first 60 minutes), systemic and local will be displayed in tabular format and stratified by dose cohort.
c. AEs will be summarized by severity and relationship to vaccine by individuals and dose cohort.
d. The proportion of volunteers with at least one local adverse event will be compared by dose cohort for each $MSP1_{42}$ vaccine serotype. Formal statistical tests will assess whether the

three cohorts differ with respect to these proportions, whether there is a dose-response relationship, and difference in proportions with respect to subsequent vaccination within a given dose cohort.

Secondary Objective 1: To determine the dose that generates the highest serum antibody levels to homologous $MSP1_{42}$ antigen at Day 42.

a. Mann-Whitney tests will be used to compare the antibody concentrations between groups. Specifically, the low (5 µg)- and medium (20 µg)-dose groups will be compared, and the medium and high (80 µg) dose groups will be compared. Each test will be performed at $\alpha = 0.025$ to control for multiple comparisons.

Secondary Objective 2: To assess and compare the duration of specific antibody response to homologous and heterologous $MSP1_{42}$ over a 12 month period.

a. Describe immunogenicity responses by vaccine and dose group, over time.
b. Individual responses will be described over time and stratified by dose cohort.
c. Antibody concentration will be measured at Days 0, 14, 28, 35, 42, 120, 180, 194, 270 and 364. To exploit the multiple measures of antibody within each subject, a longitudinal model with an appropriate covariance structure will be built to describe the antibody response over time. The model will include each of the dose groups and a term for the $MSP1_{42}$-FVO and $MSP1_{42}$-3D7 serotype. The level of antibody at Day 42 will be estimated from the resulting model collapsing over the two serotypes. Formal statistical tests will assess whether the response is monotone (low dose < medium dose < high dose) for the three doses of vaccine.
d. Various exploratory methods will be used to assess the sensitivity of the results to the assumptions in the model.
e. Analysis will be performed using longitudinal models with an unstructured covariance matrix.

Secondary Objective 3: Measure the effect of boosting at 6 months on antibody concentration.

a. The longitudinal model described above will be used to compare the level of antibody at Day 42 (post immunization 2) to the level at Day 194 (post immunization 3). Again, these comparisons will address dose response and difference between serotypes.
b. In addition, the increase in the levels of antibody between Day 28 and Day 42, and the increase between Days 180 and 194 will be estimated and compared.
c. Waning will be assessed by comparing levels of antibody at Days 270 and 364 with the level at Day 194.

The following parameters will be measured for information only:
Parameter 1: To measure the ability of the vaccine induced antibody to inhibit parasite growth as measured by the in vitro growth inhibition assay (GIA) to both FVO and 3D7 *P. falciparum* lines.

a. Graphs will display growth inhibition expressed as a percent of inhibition comparing test sera to pre-immune sera.

b. Depending on the distribution of the data, parametric or non-parametric methods will be used to compare inhibition as a function of dose, and serotype.

Parameter 2: To determine the relationship between anti-$MSP1_{42}$ antibody levels and degree of in vitro parasite growth inhibition.

a. Non-linear regression will be used to determine the goodness of fit of the data to a hyperbolic function and the ELISA value giving 50% growth inhibition will be calculated.

Should the need arise for terminating the study early, the investigative team will discuss with the SMC the reason for termination and determine which study questions can be addressed in an unbiased manner with the available data. The available data will be analyzed and interpreted in light of early termination. Deviations from the statistical plan will be reported in the study report.

9.1.2 Safety

The primary safety endpoint is the frequency and severity of vaccine-related AEs, as classified by both intensity and severity through active and passive surveillance. Separate assessments of systemic and local reactions will be performed.

9.1.3 Immunogenicity Analysis

The primary immunogenicity endpoint will be evaluation of Day 42 sera. Anti-$MSP1_{42}$ antibodies will be measured by ELISA on Days 0, 14, 28, 42, 120, 180, 194, 270, and 364 as listed in the schedule of visits (**Appendix E**). Additionally, the growth inhibition assay will be performed on Days 0, 42, 120, 180, 194 and 364 as a way to assess the biologic activity of the vaccines.

9.2 Sample Size

Based on an analysis of the human antibody responses to a number of malaria antigens that have been tested in clinical trials (the three components of Combination B [25] and RTS,S [26]), the observed coefficient of variation in the range of antibody concentrations has been found to be remarkably constant at approximately 1.2 - 1.4. Based on the distribution of antibody responses for each of the antigens in the combination B and RTS,S trials, a sample size of 10 volunteers per dose group would permit detection of at least a five-fold difference in antibody concentration between groups using a Mann-Whitney test, assuming a level of significance of 0.05 and a power of 0.80. Additionally, a group size of 10 volunteers per dose gives 0.80 probability for detecting one or more serious or severe AE that occurred with a probability of 0.15 per volunteer.

10.0 PROTECTION OF HUMAN SUBJECTS

10.1 Institutional Review Board/Ethics Committee

The Investigator will be responsible for obtaining IRB approval for the study. Before the start of the study, the appropriate documents (including the Protocol, Investigator's Brochure, Informed Consent Form, information sheets, CRFs and advertisements) will be submitted to the IRB. A copy of the study approval (including approval of the informed consent form) is to be maintained in the Investigator's study document binder and a copy will be supplied to the IND Sponsor.

During the study, the Investigator is responsible for providing the IRB with all documents subject to review (i.e., Protocol Amendments, informed consent form updates, advertisements, and any written information that may be provided to the subject). Annual reports on the progress of the study will be made to the IRBs by the Investigator in accordance with IRB guidelines and government regulations.

10.2 Informed Consent

In obtaining and documenting informed consent, the Investigator must comply with the applicable regulatory requirements, Good Clinical Practices and ethical principles. The written informed consent form must be approved by all IRBs prior to its use.

An addendum to the informed consent will be provided to volunteers in the medium dose cohort (groups B & E) and the high dose cohort (groups C & F) prior to receiving the first vaccination describing the cumulative adverse events observed in the preceding dose cohort. Volunteers will be asked to sign this addendum if they agree to continue participation in the study based on the provided information. This addendum will be submitted to each participating IRB for expedited review.

10.3 Risks

Risks to the volunteers are associated with venipuncture and with immunization. These risks are outlined below.

Female participants will be cautioned of the unknown risk of study vaccines to the fetus and will be advised to use adequate birth control methods for the duration of the study.

10.3.1 Venipuncture

Risks occasionally associated with venipuncture include pain and bruising at the site of venipuncture, lightheadedness, and syncope (rarely). Volunteers will be advised not to donate blood for 30 days after the study ends due to the amount of blood drawn during the study.

10.3.2 Immunization

Possible local vaccine reactions include pain, swelling, erythema, induration, limitation of limb movement for several days, lymphadenopathy or pruritus at the injection site. Local SC nodules, believed to be granulomatous reactions to aluminum, have been observed with use of aluminum-based adjuvants. Thus, most aluminum-adsorbed vaccines are injected IM rather than SC. Systemic reactions such as fever, chills, headache, fatigue, malaise, myalgia and joint pain may also possibly occur. Immediate hypersensitivity reactions including urticaria, anaphylaxis or other IgE mediated responses are possible as with any vaccine. As with any investigational vaccine, there is a theoretical possibility of risks about which we have no present knowledge. Volunteers will be informed of any such risks should further data become available.

10.4 Benefits

Volunteers will not receive any direct benefit from participation in this study. It is hoped that information gained in this study will contribute to the development of a safe and effective malaria vaccine.

10.5 Confidentiality

All study-related information will be stored securely at the study site. All participant information will be stored in locked file cabinets in areas with access limited to study staff. All laboratory specimens, reports, study data collection, process and administrative forms will be identified by coded number only to maintain participant confidentiality. All computer entry will be done by coded number only, and all local databases will be secured with password-protected access systems. Forms, lists, logbooks, appointment books and any other listings that link participant ID numbers to other identifying information will be stored in a separate, locked file in an area with limited access.

Participants' study information will not be released without the written permission of the participant, except as necessary for monitoring by NIAID and/or its contractors and the FDA.

10.6 Compensation

Volunteers will be paid $150 per visit during participation in the study ($2850 for 19 visits) for their time and inconvenience. Volunteers will be paid for the screening visit, if enrolled. A bonus of $1150 will be paid for completion of all visits. The total payment will be divided over the course of the study with the bonus dispensed upon completion of the trial as described in Table 3.

**Table 3: Dispersement of compensation:**	
**Study Day**	**Amount**
14	$900
42	$750
120	$150
194	$750
364	$300 + $1150 bonus
Total	$4000

11.0 REFERENCES

1. World Health Organization, Tropical Disease Research. Source: World Health Report 2002. http://www.who.int/tdr/diseases/malaria/diseaseinfo.htm
2. Good, M.F., Kaslow, D.C. & Miller, L.H. Pathways and strategies for developing a malaria blood-stage vaccine. Annu. Rev. Immunol. 16:57-87 (1998).
3. Holder A.A. & Freeman R.R. Protective antigens of rodent and human bloodstage malaria. Philos Trans R Soc Lond B Biol Sci. 307:171-7 (1984).
4. McBride J.S. & Heidrich H.G. Fragments of the polymorphic Mr 185,000 glycoprotein from the surface of isolated Plasmodium falciparum merozoites form an antigenic complex. Mol Biochem Parasitol. 23:71-84 (1987).
5. Gerold P, Schofield L, Blackman MJ, et al. Structural analysis of the glycosyl-phosphatidylinositol membrane anchor of the merozoite surface proteins-1 and -2 of Plasmodium falciparum. Mol Biochem Parasitol. 75:131-43 (1996).
6. Haldar K, Ferguson MA, Cross GA. Acylation of a Plasmodium falciparum merozoite surface antigen via sn-1,2-diacyl glycerol. J Biol Chem. 260:4969-74 (1985).
7. Holder AA, Blackman MJ, Burghaus PA, et al. A malaria merozoite surface protein (MSP1)-structure, processing and function. Mem Inst Oswaldo Cruz. 87 Suppl 3:37-42 (1992).
8. Blackman MJ, Heidrich HG, Donachie S, et al. A single fragment of a malaria merozoite surface protein remains on the parasite during red cell invasion and is the target of invasion-inhibiting antibodies. J Exp Med. 172:379-82 (1990)
9. Blackman MJ, Whittle H, Holder AA. Processing of the Plasmodium falciparum major merozoite surface protein-1: identification of a 33-kilodalton secondary processing product which is shed prior to erythrocyte invasion. Mol Biochem Parasitol. 49:35-44 (1991).
10. Chang SP, Gibson HL, Lee-Ng CT, Barr PJ, Hui GS. A carboxyl-terminal fragment of Plasmodium falciparum gp195 expressed by a

recombinant baculovirus induces antibodies that completely inhibit parasite growth. J Immunol. 149:548-55 (1992).
11. Holder AA, Freeman RR. Immunization against blood-stage rodent malaria using purified parasite antigens. Nature. 294:361-4 (1981).
12. Chauhan VS, Chatterjee S, Johar PK. Synthetic peptides based on conserved Plasmodium falciparum antigens are immunogenic and protective against Plasmodium yoelii malaria. Parasite Immunol. 15:239-42 (1993).
13. Daly TM, Long CA. A recombinant 15-kilodalton carboxyl-terminal fragment of Plasmodium yoelii yoelii 17XL merozoite surface protein 1 induces a protective immune response in mice. Infect Immun. 61:2462-7 (1993).
14. Ling IT, Ogun SA, Holder AA. Immunization against malaria with a recombinant protein. Parasite Immunol. 16:63-7 (1994).
15. Tian JH, Miller LH, Kaslow DC, Ahlers J, Good MF, Alling DW, Berzofsky JA, Kumar S. Genetic regulation of protective immune response in congenic strains of mice vaccinated with a subunit malaria vaccine. J Immunol. 157:1176-83 (1996).
16. Hirunpetcharat C, Tian JH, Kaslow DC, et al. Complete protective immunity induced in mice by immunization with the 19-kilodalton carboxyl-terminal fragment of the merozoite surface protein-1 (MSP1[19]) of Plasmodium yoelii expressed in Saccharomyces cerevisiae: correlation of protection with antigen-specific antibody titer, but not with effector CD4+ T cells. J Immunol. 159:3400-11 (1997).
17. Tian JH, Kumar S, Kaslow DC, Miller LH. Comparison of protection induced by immunization with recombinant proteins from different regions of merozoite surface protein 1 of Plasmodium yoelii. Infect Immun. 65:3032-6 (1997).
18. Kumar S, Yadava A, Keister DB, et al. Immunogenicity and in vivo efficacy of recombinant Plasmodium falciparum merozoite surface protein-1 in Aotus monkeys. Mol Med. 1:325-32 (1995).
19. Chang SP, Case SE, Gosnell WL, et al. A recombinant baculovirus 42-kilodalton C-terminal fragment of Plasmodium falciparum merozoite surface protein 1 protects Aotus monkeys against malaria. Infect Immun. 64:253-61 (1996).
20. Stowers AW, Cioce V, Shimp RL, et al. Efficacy of two alternate vaccines based on Plasmodium falciparum merozoite surface protein 1 in an Aotus challenge trial. Infect Immun. 69:1536-46 (2001).
21. Stowers AW, Chen Lh LH, Zhang Y, et al. A recombinant vaccine expressed in the milk of transgenic mice protects Aotus monkeys from a lethal challenge with Plasmodium falciparum. Proc Natl Acad Sci U S A. 99:339-44 (2002). Epub 2001 Dec 18.
22. Singh S, Kennedy MC, Long CA, et al. Biochemical and immunological characterization of bacterially expressed and refolded Plasmodium falciparum 42-kilodalton C-terminal merozoite surface protein 1. Infect Immun. 71:6766-74 (2003).

23. Cody, C.L., Baraff, L.J., Cherry, J.D., Marcy, S.M. & Manclark, C.R. Nature and rates of adverse reactions associated with DTP and DT immunizations in infants and children. Pediatrics 68:650-660 (1981).
24. Merck and Co., I. Recombivax HB Product Insert. http://www.whale.to/m/recombivax_hb.html. 1999. 2-14-2002.
25. Saul, A., Lawrence G., Smillie A., et al. Human Phase 1 vaccine trials of 3 recombinant asexual stage malaria antigens with Montanide ISA 720 adjuvant. Vaccine 17:3145-3159 (1999).
26. Stoute, J.A., Slaoui, M., Heppner, D.G., et al. A preliminary evaluation of a recombinant circumsporozoite protein vaccine against Plasmodium falciparum malaria. RTS,S Malaria Vaccine Evaluation Group. N Engl J Med. 336:86-91 (1997).
27. Cody, C.L., Baraff, L.J., Cherry, J.D., Marcy, S.M. & Manclark, C.R. Nature and rates of adverse reactions associated with DTP and DT immunizations in infants and children. Pediatrics 68:650-660 (1981).
28. Merck and Co., I. Recombivax HB Product Insert. http://www.whale.to/m/recombivax_hb.html. 1999. 2-14-2002.
29. Rotman HL, Daly TM, Clynes R, Long CA. Fc receptors are not required for antibody-mediated protection against lethal malaria challenge in a mouse model. J Immunol 161(4):1908-12 (1998).
30. Vukovic P, Hogarth PM, Barnes N, Kaslow DC, Good MF. Immunoglobulin G3 antibodies specific for the 19-kilodalton carboxyl-terminal fragment of Plasmodium yoelii merozoite surface protein 1 transfer protection to mice deficient in Fc-gammaRI receptors. Infect Immun. 68(5):3019-22 (2000).
31. May JC, Progar JJ, Chin R. The aluminum content of biological products containing aluminum adjuvants: determination by atomic absorption spectrometry. J Biol Stand 12:175-183 (1984).
